# bindNode24: Competitive binding residue prediction with 60 % smaller model

**DOI:** 10.1016/j.csbj.2025.02.042

**Published:** 2025-03-11

**Authors:** Kyra Erckert, Franz Birkeneder, Burkhard Rost

**Affiliations:** a TUM School of Computation, Information and Technology, Bioinformatics & Computational Biology - i12, Boltzmannstr. 3, Garching, Munich 85748, Germany; b TUM Graduate School, Center of Doctoral Studies in Informatics and its Applications (CeDoSIA), Boltzmannstr. 11, Garching 85748, Germany; c Institute for Advanced Study (TUM-IAS), Lichtenbergstr. 2a, Garching, Munich 85748, Germany; d Germany & TUM School of Life Sciences Weihenstephan (TUM-WZW), Alte Akademie 8, Freising, Germany

**Keywords:** Binding residue prediction, Protein language model, Machine learning, Graph neural networks, Embeddings, Binding residues, Protein binding

## Abstract

Many proteins function through ligand binding. Yet, reliable experimental binding data remains limited. Recent advances predict binding residues from sequences using protein Language Model embeddings. The AlphaFold Protein Structure Database, which has reliable 3D structure predictions from AlphaFold2, opens the way for graph neural networks that predict binding residues. Here, we introduce *bindNode24*, a new method using Graph Neural Networks to predict whether a residue binds to any of three ligand classes: small molecules, metal ions, and nucleic macromolecules. Compared to state-of-the-art, this approach reduces the number of free parameters by almost 60 % at similar performance. Our findings also suggest that secondary and tertiary structure features from *AlphaFold2* are easy to integrate into protein function prediction tasks that previously solely relied on protein Language Model embeddings.

## Graphical Abstract

**Figure:**
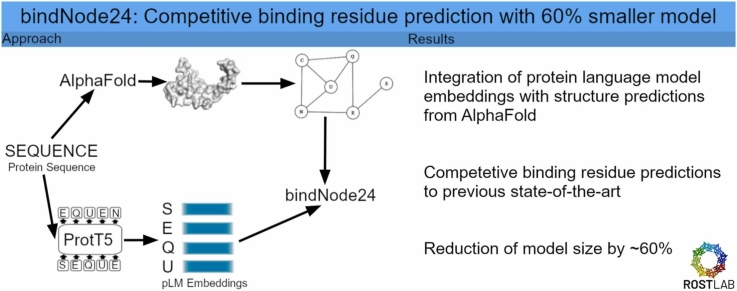

## 1 Introduction

### 1.1 Reliable binding residue predictions are important

Proteins play a fundamental role in biological processes, and understanding their function is crucial for deciphering cellular mechanisms [1]. Many proteins function by binding to specific ligands, such as small molecules, metal ions, or DNA/RNA macromolecules [2]. Identifying the residues that bind ligands is essential because these residues directly influence enzymatic activity, molecular recognition, and protein interactions. Well-calibrated, reliable ligand binding predictions can provide valuable insights into protein function and might assist in the annotation of newly sequenced proteins and/or in the design of targeted therapeutics [3].

Reliable experimental binding data remains limited by demands in time and resources. For instance, over almost two years, non-redundant high-resolution annotations [4] for residue binding of any non-protein substrate in any organism amounted to just about 1014 proteins, but most dramatically, only for 46 of those (<5 %), the annotations were novel, while the other 95 % were just confirmations of what had already been known [5]. While such confirmations may be biologically relevant (e.g., establishing in mice that something binds that had been shown for flies), they do not widen the intrusion into the unknown. Computational predictions offer a cost-efficient alternative. Protein Language Models (pLMs) have recently emerged as powerful tools for predicting binding residues from sequence embeddings, reaching levels of performance almost as reliable as low-resolution experiments at a fraction of the cost [5], [6], [7].

These models are instrumental in addressing the challenge posed by exponentially growing protein sequence databases [8]. Given this rapid growth, adding new protein sequences vastly outpaces the ability to experimentally determine protein function for those. As more sequences are added to databases like UniProt[8], automated functional annotation becomes central to keeping up with the exponential growth. Despite the successes of pLMs in bridging the sequence-annotation gap (the difference between the number of proteins of known sequence and those with experimentally supported annotations), these approaches usually lack the integration of structural information, which is crucial for protein function.

### 1.2 Understanding binding requires integrating structure

Protein function is inherently tied to intrinsic details of 3D structure. This includes structural features such as binding pocket geometry and surface accessibility. Two proteins with similar sequences may differ in their binding properties due to minor conformational differences. Conversely, proteins with unrelated sequences might adopt similar 3D structures and share functional characteristics. Therefore, the integration of structural information could enhance binding residue predictions. Approaches such as DeepSite [9], GraphBind [10], ECOD [11], and FTMap[12] integrate protein structure to boost performance.

The first bilingual pLM, ProstT5 [13] tries to integrate structural information into the language model framework by integrating 3Di tokens [14] during training. However, this indirect way of 3D structure projected onto a string so successful for structure comparisons by Foldseek [14], has not yet improved the success of transfer-learning (using embeddings as input to subsequent supervised prediction tasks such as the protein structure) as we may have hoped [15]. Other pLMs use structural information in different ways (e.g., SSA [16], Saprot [17], PST [18], ESM3 [19]). *AlphaFold2* [20] has been making high-quality structure predictions available through the AlphaFold Protein Structure Database (AFDB) [21]. This provides an opportunity to leverage protein topology for improved binding prediction instead of relying on sequence-only approaches.

### 1.3 GNNs exploit structure to predict protein-ligand binding

Graph Neural Networks (GNNs) excel at capturing local structural details by using neighborhood graphs to model spatial relationships between amino acids. This can be particularly useful to enforce the rotational and translational invariance observed in proteins.

Recent methods, including PGpocket[22], GraphBind [10], SiteRadar [23], LigBind [24], and NABind[25], have effectively demonstrated the power of GNNs. However, many recent solutions remain limited to binary binding/non-binding predictions or can only predict binding residues for a single ligand type.

To address this gap, we developed *bindNode24*, a novel binding-residue prediction method integrating observed or predicted information about inter-residue distances into a GNN. By leveraging structures predicted by Colabfold [26], bindNode24 captures spatial relationships between protein residues; it distinguishes three different types of ligands: small molecules, nucleic acids, and metal ions.

Compared to sequence-only models such as bindEmbed21DL [5], our method, bindNode24, reached similar levels of accuracy at a substantial reduction in trainable parameters. This highlights its efficiency and suitability for large-scale protein function annotation and drug discovery applications. Overall, the GNN-based integration of structural information improved several aspects of the prediction, underscoring this combination's relevance for protein function annotation.

## 2 Methods

### 2.1 Data set

To ensure a fair comparison to bindEmbed21DL [5], we used the same data set comprising 1314 proteins with reliable experimental binding annotations from BioLip [4]. We maintained the original split into 1014 sequences for cross-training (*DevSet1014*), using the same five splits as bindEmbed21DL and 300 for the final performance evaluation (*TestSet300*). We enriched the dataset with structural information by generating protein 3D structure predictions with Colabfold [26] (1310 protein structures).

We excluded four sequences (Table S1, Supporting Online Material - SOM) due to length mismatches between the original protein sequence and the available predicted protein structure. Removing these four resulted in 1010 sequences for cross-training (*DevSet1010* with 13,950 binding and 156,231 non-binding residues; Table S3) and 300 for evaluation (*TestSet300* with 5869 binding and 56.820 non-binding residues; Table S3).

### 2.2 Embeddings

We used the following five pLMs to generate embeddings: The transformer-based ProtT5 [27], ProtBert [27], Ankh [28], ProstT5 [13] and ESM-2 (3B) [29]. We compared the performances of all embeddings using the same architecture. In the following, we try to motivate the inclusion of each pLM. ***ProtBert*** [27]***:*** As an early-generation pLM, it provides a good baseline for the performance of a simple transformer-based solution [27]. While outperformed for many tasks by more recent pLMs [30], it has been used in trendsetting solution [31], [32]. ***ProtT5*** [27]***:*** Another early-generation pLM that has repeatedly been shown to achieve state-of-the-art prediction performance in transfer learning [33]. Furthermore, per-protein embeddings of this model for some reference genomes and model organisms are easily accessible through UniProt (https://www.uniprot.org/help/embeddings). ***Ankh*** [28]***:*** A more recent pLM optimized to be successful across a wide range of transfer learning tasks [28]. Providing both a small (Ankh-base) and a large version (Ankh) is one way to capture the influence of model size on downstream performance. This was particularly relevant for our study to generate a lean solution (the smallest model that is good enough). ***ESM-2 (3B)*** [29]***:*** is possibly the most widely used pLM. This specific model provides a good tradeoff between size and downstream prediction performance on diverse tasks. ***ProstT5*** [13]***:*** This is the first bilingual pLM explicitly modeling the structure as input and output of the model through the 3Di tokens introduced by Foldseek [14]. ProstT5 is trained simultaneously on sequence and structure and learned to translate from one to the other in both directions [13]. For us, ProstT5 served as a simple, resource-efficient proxy for a pLM that included structural information. An additional advantage was that the adaptations for ProstT5 after using ProtT5 were minimal. Possible alternatives somehow using structural information to generate pLMs such as SSA [16], ProtTucker [34], PST [18], Saprot [17], or ESM3 [19] required more effort to test.

We also evaluated DistilProtBert[35] and OntoProtein[36] which are size-reduced or differently trained embedding alternatives. However, in our hands, these two did not perform competitively (SOM: Tables S10, S19) and were therefore excluded from the main analysis.

### 2.3 Protein feature extraction

For all proteins with binding data, we generated Colabfold [26] structure predictions and computed structural features through DSSP [37]. In particular, we computed relative residue position, secondary structure, phi and psi angles, solvent accessibility, and relative indices and energies of side chain groups. We used the 3D coordinates of C_α_-atoms to calculate 2D inter-residue distance maps for all residue pairs. We tested different Ångstrøm cutoffs (1–20 Å = 0.1–2 nm) to build connectivity graphs from the distance map. As a baseline, we used the models with the 4 Å cutoff, which contains only edges representing the protein backbone. This can be considered the graph representation of the amino acid string without any additional structural information.

### 2.4 Input features

We converted the actual residue positions into relative values between 0 and 1, dividing by sequence length. Per-residue assignments of secondary structures were one-hot encoded into eight bins based on the computed DSSP structural classes, phi and psi angles normalized to values between 0 and 1 by dividing by 360° and relative indices and energies of side chain groups were min-max normalized on a per-protein level. The DSSP algorithm implemented in biopython already normalized solvent accessibility values and, therefore, did not require any additional processing [38]. We constructed 20-dimensional DSSP feature vectors from these normalized values for each amino acid. Per-residue embedding representations were generated as additional feature vectors (more details in SOM section “Pseudocode snippet: DSSP feature normalization”).

### 2.5 Prediction method

We compared four different types of graph neural networks (GNNs) inputting ProtT5 [27] embeddings alone, or inputting ProtT5 and DSSP [37] features (below) on DevSet1014. We integrated the predicted structure to set up the graph neural networks (GNNs). Edges were weighted for message passing based on the distance in the protein structure (3D-close residues: high weights, 3D-distant residues within the cutoff: low weights). To limit the space of possible hyper-parameters, we exclusively used the best-performing model (SAGEConv) from this comparison to select the best-performing pLM; this was ProtT5. Finally, we evaluated the performance of the SAGEConv model using ProtT5 [27] embeddings on the test set (TestSet300) and compared it to state-of-the-art (SOTA) methods. As our final model, we refer to this one as *bindNode24*.

### 2.6 Models

All models output for each amino acid probabilities for the three types of binding residues (S: small, N: nuclear, M: metal). Residues predicted with values exceeding 0.5 (≥0.5) in class C were considered to bind C; residues were considered non-binding if all three class predictions remained below the prediction threshold.

We applied class weights to our model during training to address the strong class imbalance in our dataset, following the approach from the previous work on bindEmbed21DL [5]. The class weights were specifically set to 8.9, 7.7, and 4.4 for metal, nucleic, and small binding residues, respectively. These class weights penalized misclassification of the minority class binding more than non-binding.

For the embedding comparisons, we adjusted the input dimensions to those enforced by each pLM, e.g., the SAGEConv model (below) trained on *Ankh*-base embeddings input a dimension of 768 instead of 1024 (for simplicity, we use d=N to signify the input dimension in the following). The differences between the models were as follows.

***GCNConv*** describes a graph convolutional network (GCN) consisting of two convolutional layers with a relu activation function and 0.7 dropout. The features for each node in the first layer were d= 1024 for ProtT5 [27] and were reduced to 128 node features for the second convolutional layer. For the model using DSSP features as additional input, the input dimension increased from 1024 to 1044, concatenating the 20 DSSP features to the per-residue embeddings.

***SAGEConv*** consisted of two GraphSAGE [39] layers with a leaky relu activation and 0.7 dropout. The features for each node in the first layer were d= 1024 for ProtT5, reduced to d= 128 in the second GraphSAGE layer. The input dimension for this model also increased to d= 1044 for the models using DSSP features.

***SAGEConvMLP*** consisted of a single GraphSAGE layer followed by two fully connected layers with a leaky relu activation after the GraphSAGE layer, a relu activation after the first fully connected layer, and 0.7 dropout. Input to the first layer was d= 1024 for ProtT5, reduced to d= 128 for the first fully connected and d= 32 for the second fully connected layer. For the model including DSSP features, the input dimension of the first fully connected layer increased to 148 and the input for the second fully connected layer to 37. The 20 DSSP features were concatenated to the output of the GraphSAGE layer.

***SAGEConvGATMLP*** consisted of a GraphSAGE layer with d= 1024 for ProtT5 and 128 for input and output, respectively. Parallel to this GraphSAGE layer, a second GraphSAGE layer extracted 128 features from either 1024 embedding inputs (non-DSSP model) or 20 DSSP inputs (DSSP model). The output of the second GraphSAGE layer was further processed by two GATv2 [40] layers, halving the output dimension in each layer. The 128-dimensional output from the first GraphSAGE layer was concatenated with the 32-dimensional output from the final GATv2 layer. This 160-dimensional feature vector was sequentially input into two consecutive fully connected layers. As for the other models, we used leaky relu and relu activation functions and a dropout of 0.7.

### 2.7 Performance measures

We evaluated performance through a variety of measures that are commonly used for binding residue prediction. For simplicity, we used the following standard annotations: True positives (TP) were correctly predicted as binding residues, while false positives (FP) were residues without any binding annotations incorrectly predicted to be involved in binding. True negatives (TN) were correctly predicted as non-binding, and false negatives (FN) were residues annotated to be binding and predicted as non-binding. We accept incorrect estimates originating from the fact that “not observed” does not always imply “not existing,” i.e., some of the FN might be binding residues that have not been experimentally confirmed. Based on these classifications for the per-residue predictions, we calculated Matthews Correlation Coefficient (MCC, Eq. (1)), F1 score (Eq. (5)), precision (Eq. (2)), recall (Eq. (3)), and accuracy (Eq. (4)) separately for each type of binding residue.

(1) $$\mathrm{MCC}=\frac{\mathrm{TP}\times \mathrm{TN}-\mathrm{FN}\times \mathrm{FP}}{\sqrt{\left(\mathrm{TP}+\mathrm{FP})\times (\mathrm{TP}+\mathrm{FN})\times (\mathrm{TN}+\mathrm{FP})\times (\mathrm{TN}+\mathrm{FN}\right)}}$$

(2) $$\mathrm{Precision}=\frac{\mathrm{TP}}{\mathrm{TP}+\mathrm{FP}}$$

(3) $$\mathrm{Recall}=\;\frac{\mathrm{TP}}{\mathrm{TP}+\mathrm{FN}}$$

(4) $$\mathrm{Accuracy}=\;\frac{\mathrm{TP}+\mathrm{TN}}{\mathrm{TP}+\mathrm{FP}+\mathrm{FN}+\mathrm{FP}}$$

(5) $$F1=2\frac{\mathrm{Precision}x\mathrm{Recall}}{\mathrm{Precision}+\mathrm{Recall}}$$

For each performance measure, we computed confidence intervals (CI) by calculating the performance measure for each protein individually and then computing the mean over the resulting distribution and symmetric 95 % CI, assuming a normal distribution of the performance values.

Our presentation mostly focused on the F1 score as the primary performance metric, as it summarizes both *Precision* and *Recall* in a single number. All other performance measures were confined to the SOM as they did not provide additional insights.

### 2.8 Comparison to other methods

We compared our new method to two other methods, namely *bindEmbed21DL* [5] and *GRaSP* [41] (SOM Table S4 partially motivates this restriction). The base comparison is with bindEmbed21DL [5] from which we copied most of the design and solution but added structure through GNNs. bindEmbed21DL used a shallow two-layer Convolutional Neural Network (CNN) [5] and - having been trained and tested in the same way – it is directly comparable. GRaSP [41] predicts binding residues through graph-based residue representations of residues and their neighborhood. As it also incorporates structural information – but in the traditional solution of focusing on expert-selected features rather than pLM embeddings – it provides an informative comparison. As an additional benefit, the authors have shown that GRaSP performed favorably to many other SOTA methods, allowing readers to triangulate indirectly.

### 2.9 Runtime evaluation

To compare inference efficiency between bindNode24 and bindEmbed21DL [5], we systematically measured runtime on randomly generated protein sequences of varying lengths. Specifically, we generated 1000 random sequences for each sequence length from 50 to 1000 residues in increments of five. For each generated set of sequences, we recorded the total runtime required by each model to generate binding residue predictions.

To ensure a fair comparison, the recorded runtimes only measured model inference time, excluding pre-processing, data loading, and post-processing steps. We conducted each experiment on the same hardware (32 GB RAM, 3.00 GHz 64-bit processor).

### 2.10 Availability of method, data, and materials

The source code of our method, the models trained, and the dataset are all freely available as a GitHub repository (https://github.com/erckert/bindNode24).

## 3 Results & discussion

### 3.1 Effect of structural features on binding prediction

We expect structural information such as predicted 3D structure from AlphaFold2 [20], ColabFold [26], or even 1D projections from DSSP [37] to improve binding predictions by providing information on biophysical constraints. Minimally, binding sites need to be accessible to ligands, i.e., the information on whether a residue is close to the surface should matter. Binding pockets also need to fit the ligand.

Contrary to this expectation, using DSSP features input in addition to the embeddings did not significantly improve performance (measured by F1, Eq. (5)) for our GNN-based models dubbed *GCNConv* and *SAGEConv* (Tables S6-S7, S11-S12, S15-S16, S20-S21). In contrast, DSSP features numerically increased F1 for more complex models such as *SAGEConvMLP* and *SAGEConvGATMLP* (Tables S8-S9, S13-S14, S17-S18, S22-S23). These findings suggested that the degree to which structural features improved the binding prediction depended on the GNN architecture. However, although DSSP input improved *SAGEConvMLP* and *SAGEConvGATMLP,* these two were outperformed by the *SAGEConv* models (Tables S6-S23).

**Fig. 1:**
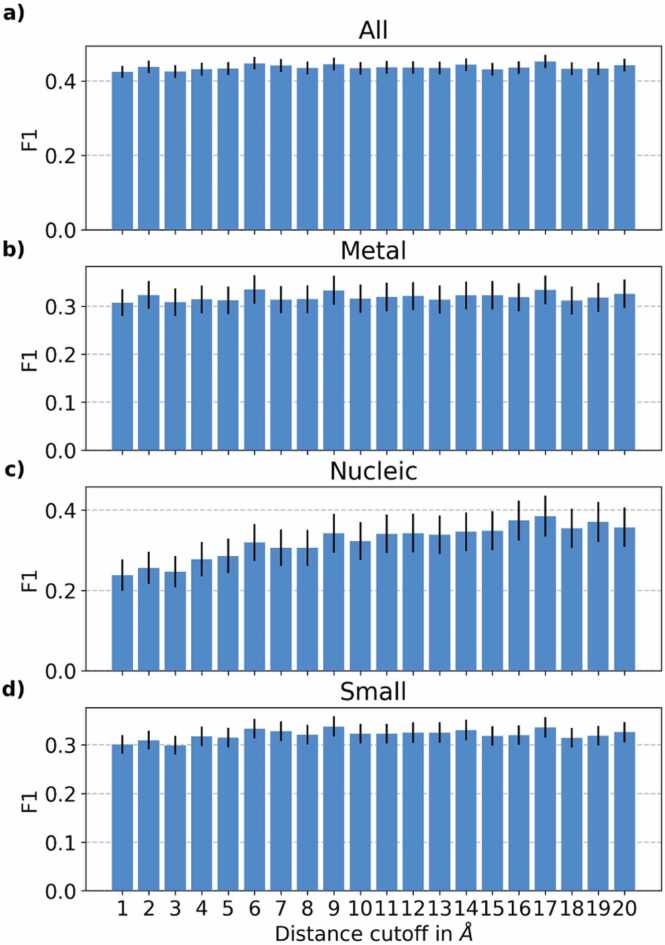
Performance vs. distance threshold. The SAGEConv model creates graphs between residue pairs depending on their spatial proximity. We tested the effect of different distance thresholds (x-axes: from 1 to 20 Å) on F1 (Eq. (5)) for four different tasks (on validation set): a) binary binding vs. non-binding, b) metal-binding, c) DNA/RNA-binding (nucleic acid-binding), d) small-molecule binding. Only the prediction of nucleic acid-binding appeared to perform better for higher thresholds (up to ∼17 Å). Error bars mark 1.96 % standard errors, i.e., the 95 % confidence interval (CI).

Overall, different structural features affected binding predictions differently. While DSSP-derived features provided limited benefits, the distance threshold (T) for building protein graphs mattered in the following sense. Evaluating the effect of different distance thresholds on the binding residue class, we found that the optimal distance threshold differed between binding classes (all, metal, small-molecule, nucleic acid; Fig. 1). While the 17 Å threshold performed well for *SAGEConv* for all classes, small molecule-binding reached numerically higher F1 at 9 Å (Fig. 1d, Tables S7, S12, S16, S21). Which threshold performed best also depended on the model architecture: *GCNConv* consistently achieved the numerically highest F1 for T ≤ 4 Å (Tables S6, S11, S15, S20); all other models required T > 5 Å for numerically highest performance. Many models reached peak performance for T > 10 Å (Tables S7-S9, S12-S14, S16-S18, S21-S23). These results highlight the importance of model-specific hyper-parameter selection when integrating structure. Due to achieving numerically and often also statistically significantly highest performances for *SAGEConv*, we focused on this model from this point onwards.

### 3.2 Effect of pLM on binding prediction

We tested six pLMs and found that only the simpler *Ankh-base* [28] and *ProtBert* [27] (not to confuse with ProtBERT [42]) performed significantly worse. The F1 performance for the other four (ProstT5 [13], ProtT5 [27], ESM-2/3b [29] and Ankh [28]) did not differ within the 95 % confidence interval (CI), i.e., differed by fewer than ± 1.96 standard errors (other performance measures behaved alike, Table S10).

**Fig. 2:**
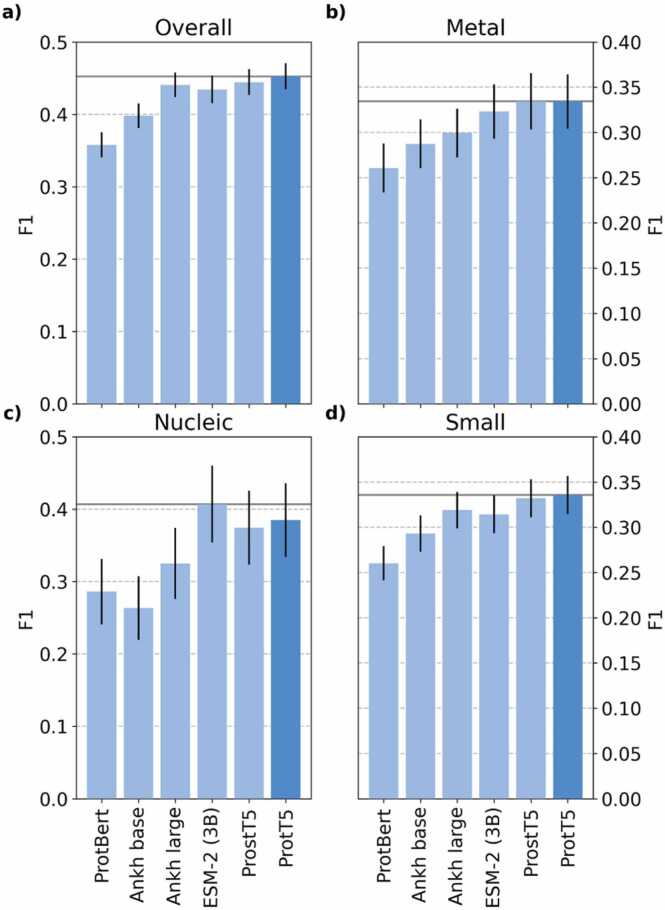
Performance vs pLM. We tested the effect of six different pLMs (x-axes) on the performance of SAGEConv (F1 performance for validation set): a) binary binding vs. non-binding, b) metal-binding, c) DNA/RNA-binding (nucleic acid-binding), d) small-molecule binding. Four pLMs reached similar performance levels (alphabetically: Ankh large [28], ESM-2 [29] (3B parameter model), ProstT5 [15], and ProtT5 [27]) while – for some classes - outperforming simpler pLMs such as Ankh small [28] and ProtBert [27]. ProtT5 reached numerically the highest F1 except for DNA/RNA-binding for which ESM-2 was highest. However, these differences were not statistically significant at the 95 % confidence interval (error bars mark 1.96 % standard errors, i.e., the 95 % confidence interval (CI)). Horizontal grey lines indicate the highest value for each ligand type. Dark blue bars indicate our final embedding choice for bindNode24.

Notably, ProstT5, a model specifically designed to integrate structural information, did not improve over its progenitor, ProtT5 (Fig. 2). This suggested that directly incorporating structural information into embeddings is not the right solution, that ProstT5 did not find the optimal way for this task to do it, or that the ProstT5 integration – or any other – may not capture information orthogonal to using structure as constraint for the GNN.

### 3.3 Impact of AlphaFold2 confidence on binding prediction

Predicted structures have varying levels of confidence measured by the predicted local distance difference test (plDDT) [20]. Building a predictive model on top of uncertain inputs could propagate or magnify uncertainty. Surprisingly, the Pearson correlation between the AlphaFold2-plDDT and the predicted binding residue probabilities were consistently low (Fig. S7). This suggested that less accurately predicted structure did not lead to less accurate binding residue predictions. This might originate from the reliance on neighborhood-based graph representations rather than on precise atomic coordinates in the GNNs.

### 3.4 Confidence correlates with correctness

Finally, as already observed in bindEmbed21DL, the prediction strength of *bindNode24*, i.e., its predicted reliability, also correlates with performance: more strongly predicted residues are more often predicted correctly (Fig. S3). For example, 19 % of all residues observed in binding were predicted with probability > 0.8, and for these, precision reached 77 % (Fig. S4). At thresholds in the predicted reliability above 0.95, precision rose above 87 % while maintaining a recall of 16 %. This correlation strengthens the utility of bindNode24 by giving experimentalists a reliability indicator of the individual predictions.

### 3.5 Comparison to other methods

**Fig. 3:**
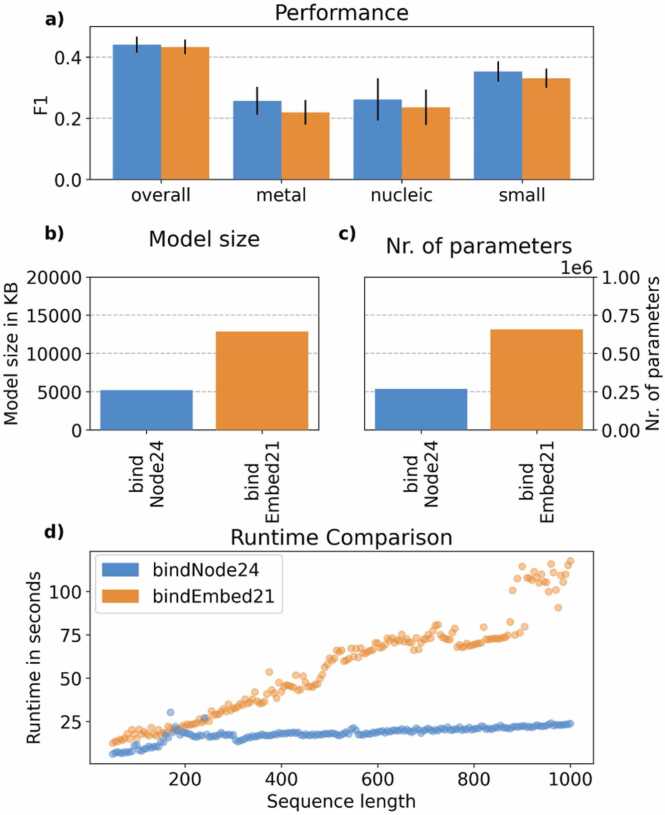
Embedding-based binding prediction with and without structure. Both bindNode24, the method introduced here, and *bindEmbed21DL*[5] primarily use ProtT5 embeddings as input, but *bindNode24* builds a GNN from Colabfold [26] structure predictions. (a) While the structure-based solution reached numerically higher accuracy (here F1, Table S5 for other measures), differences were not statistically significant. Structural information allowed *bindNode24* to reduce the model size (b) and the number of trainable parameters (c). (d) The leaner model translated directly to runtime savings: Each point represents the summed-up runtime for 1000 randomly generated sequences of a given length (from 50 to 1000 residues in increments of 5). Error bars mark 1.96 % standard errors, i.e., the 95 % confidence interval (CI).

Our final method, dubbed *bindNode24* (SAGEConv, ProtT5 embeddings, 17 Å cutoff, no DSSP features), reached numerically higher performance (F1: Fig. 3a, Table S5) than *bindEmbed21DL* [5], with a significant reduction in model parameters by 60 % (Fig. 3b, c). The parameter reduction demonstrated the efficiency of our graph-based approach. The key improvement originated from the ability of the structure-based GNN, *bindNode24*, to correctly classify non-binding residues, which explains its overall performance gains (Figs. S1, S2). We investigated ROC curves (Fig. S5) and PR (precision-recall) curves (Fig. S6) to also show performance for not only a single but many different thresholds for the binary decision binding/non-binding given extremely imbalanced data (approximately 90 % of residues are non-binding). These revealed that both the sequence-only solution bindEmbed21DL and the structure-based GNN bindNode24 performed similarly throughout (Figs. S5, S6).

*GRaSP*[41], is another graph-based method that models residue neighborhoods to predict binding sites. In our hands, *bindNode24* reached statistically significantly higher values than *GRaSP* in terms of F1 and numerically higher performances for MCC (Table 1, Table S5), highlighting its ability to balance precision and recall effectively. In contrast, *GRaSP* seems to focus on a different balance between precision and recall, reaching a higher precision (precision: 57 %, recall: 30 %, Table 1, Table S5). Notably, suppose we select the cutoff for bindNode24 that achieves a precision of approximately 57 % for the binary binding/non-binding prediction task. In that case, the corresponding recall drops to around 39 %, as indicated by our precision-recall curves on TestSet300 (Fig. S6).

**Table 1:** Performance Comparison of bindNode24 and GRaSP. *.

**Performance Measure**	**bindNode24**	**GRaSP**
MCC	0.42 ± 0.03	0.37 ± 0.03
F1	44 ± 3	36 ± 3
Precision	50 ± 3	57 ± 4
Recall	51 ± 4	30 ± 3
Accuracy	90 ± 1	91 ± 1

* Performance metrics for bindNode24 and GRaSP were evaluated on TestSet300. Metrics include Matthews Correlation Coefficient (MCC), F1 score, Precision, Recall, and Accuracy, with 95 % confidence intervals reported (1.96 % standard errors). While GRaSP [41] achieves higher precision, bindNode24 demonstrates superior F1 score, recall, and MCC, indicating a better balance between sensitivity and specificity.

The downloadable results from GRaSP focus on the binary prediction of binding/non-binding. In contrast, *bindNode24* adds separate predictions for three classes of ligands on top of the binary results.

### 3.6 Efficiency in model size and runtime

Using predicted structure through GNNs enables *bindNode24* to reduce the number of trainable parameters by 60 % (Fig. 3c) at similar performance (Fig. 3a). This directly translates into shrinking the size of the model substantially (Fig. 3b: from 12.86MB to 5.2MB). Consequently, the full training of *bindNode24* on data set *DevSet1010* using pre-computed embeddings and 5-fold cross-validation took about 30 minutes on consumer-grade hardware (32 GB RAM, 3.00 GHz 64-bit processor). With this, our approach is contrary to the recent trends observed in, e.g., natural language processing (NLP), where models tend to increase in size to get better. Our model suggested bigger is not always better: smaller models can match the performance of larger counterparts if a suitable architecture is chosen, even without tapping into LoRA-type fine-tuning [33].

Faster training helped us to save resources and translates to the same benefit every time users apply this method (Fig. 3d). The results clearly demonstrated that, especially for longer sequences, *bindNode24* will outpace *bindEmbed21DL* in terms of runtime efficiency. Thus, our architecture optimization reduced model size and the number of trainable parameters, leading to significantly faster inference time.

Considering the increasing emphasis on computational sustainability of artificial intelligence (AI) [43], [44], [45], these improvements may be highly relevant. Furthermore, small models help level the playing field by reducing the demand on compute resources for users [46]. This allows a more diverse range of researchers to contribute to AI developments in bioinformatics in the future. In addition to the previous aspects, smaller models tend to be less likely to be over-trained. Despite careful cross-validation, deep learning has made the avoidance of over-estimating performance even more challenging than it already was 30 years ago, as amply demonstrated by benchmarks such as CASP [47], CAFA [48], CAID [49] or the ProteinGym [50] (compare, in particular, the drop in the relative ranking of methods between different data sets/snap-shots).

### 3.7 Graph-based solutions ease the integration of structural information

While our solution toward integrating structural information did not improve accuracy significantly, the slight numerical improvement was consistent across all four binding classes (Fig. 3a). The use of GNNs enables the use of protein connectivity graphs to process structural information directly. This approach demonstrated the ease of integrating structural features, which is likely applicable to other protein prediction tasks. A limitation of this work is that we focused on using the connectivity graph for each protein. In contrast, AlphaFold2 internally uses a rich representation of MSAs and structural templates in its Evoformer modules [20]. These representations could be used as high-dimensional edge attributes to integrate additional information if made available.

## 4 Conclusions

We proposed a solution to integrate structural features into graph-based approaches for binding residue prediction. Some previous results challenged the intuition that 3D structure should improve predictions [51]. While the addition of structural per-residue features such as secondary structure and solvent accessibility (from DSSP [37]) helped in some cases, it neither consistently nor significantly improved. Overall, however, the concept of turning predicted 3D structure into 2D distance maps used as thresholds for the GNN succeeded, although only nucleic acid-binding prediction benefited from using large (>5 Å) distance thresholds (Fig. 1). Four of the six pLMs tested reached a relatively similar level of performance (Fig. 2). Our new model *bindNode24* reached a similar level (F1 in Fig. 3a, additional performance measures in Table S5) as the previous state-of-the-art model *bindEmbed21DL* [5]. Remarkably, this was possible while reducing trainable parameters by approximately 60 %. This reduction highlights the efficiency of our approach; it will significantly reduce the computational resources and time needed by users—good news given the environmental and accessibility concerns around AI.

Overall, our results underline the potential of GNNs for efficient and resource-conscious protein function prediction. Although structural information did not significantly improve performance, graph-based approaches provide a flexible platform for future integration of richer structural data. This adaptability may render our approach valuable to future protein function prediction approaches, promoting inclusion by enabling researchers with limited computational resources to perform and develop high-quality protein analysis.

## Funding

This work was supported by a grant from the BMBF (German Ministry for Education and Research; grant number 031L0168).

## CRediT authorship contribution statement

**Rost Burkhard:** Writing – review & editing, Writing – original draft, Supervision, Funding acquisition. **Birkeneder Franz Josef:** Writing – original draft, Validation, Software, Methodology, Investigation, Formal analysis, Data curation, Conceptualization. **Erckert Kyra Yasmin:** Writing – review & editing, Writing – original draft, Visualization, Validation, Supervision, Software, Project administration, Methodology, Investigation, Formal analysis, Conceptualization.

## Declaration of Competing Interest

The authors declare that they have no competing interests.
