# Supplementary material for: bindNode24: Competitive binding residue prediction with 60 % smaller model
Source: Comput Struct Biotechnol J. 2025 Mar 11;27:1060–6. doi: 10.1016/j.csbj.2025.02.042 (PMC11957672; doi:10.1016/j.csbj.2025.02.042)
Supplement: Supplementary file 1 — Supplementary material [file mmc1.docx]

Supporting Online Material (SOM) for:
bindNode24: Competitive binding residue prediction with 60% smaller model

Kyra Erckert, Franz Birkeneder & Burkhard Rost

# Table of Contents for SOM

[Table of Contents for SOM 1](#_Toc190271437)

[Short Description of Supporting Online Material 2](#_Toc190271438)

[Materials and Methods 4](#_Toc190271439)

[Table S1: Proteins that did not yield predicted structures. 4](#_Toc190271440)

[Table S2: Per-residue embedding dimensions. 4](#_Toc190271441)

[Table S3: Dataset distributions. 5](#_Toc190271442)

[Pseudocode snippet: DSSP feature normalization. 5](#_Toc190271443)

[Additional performance measures: CovOneBind, True Positive Rate, and False Positive Rate. 7](#_Toc190271444)

[Table S4: Methods excluded for comparison and reason for exclusion 7](#_Toc190271445)

[Additional Results 8](#_Toc190271446)

[Test Performances 8](#_Toc190271447)

[Table S5: Comparison of bindEmbed21DL, bindNode24 and GRaSP Performances on TestSet300 8](#_Toc190271448)

[Figure S1: Predicted probabilities of bindNode24 and bindEmbed21DL on TestSet300 9](#_Toc190271449)

[Figure S2: Heatmaps of bindNode24 and bindEmbed21DL predictions on TestSet300 10](#_Toc190271450)

[Figure S3: Heatmaps of bindNode24 and bindEmbed21DL reliabilities on TestSet300 11](#_Toc190271451)

[Figure S4: Precision, Recall and CovOneBind curves of bindNode24 and bindEmbed21DL on TestSet300 12](#_Toc190271452)

[Figure S5: Receiver Operating Characteristic (ROC) Curves of bindNode24 and bindEmbed21DL on TestSet300 13](#_Toc190271453)

[Figure S6: Precision-Recall (PR) Curves of bindNode24 and bindEmbed21DL on TestSet300 14](#_Toc190271454)

[Figure S7: Relationship between AlphaFold-predicted structure confidence (plDDT) and bindNode24 predictions 15](#_Toc190271455)

[Training Performances 16](#_Toc190271456)

[Without DSSP features 16](#_Toc190271457)

[Table S6: Average training performances of GCNConv on different distance cutoffs with 5-fold cross-validation. 16](#_Toc190271458)

[Table S7: Average training performances of SAGEConv on different distance cutoffs with 5-fold cross-validation. 19](#_Toc190271459)

[Table S8: Average training performances of SAGEConvMLP on different distance cutoffs with 5-fold cross-validation. 21](#_Toc190271460)

[Table S9: Average training performances of SAGEConvGATMLP on different distance cutoffs with 5-fold cross-validation. 23](#_Toc190271461)

[Table S10: Embedding comparison on SAGEConv model (cutoff: 17Å) with 5-fold cross-validation. 25](#_Toc190271462)

[With DSSP features 26](#_Toc190271463)

[Table S11: Average training performances of GCNConv on different distance cutoffs with 5-fold cross-validation. 26](#_Toc190271464)

[Table S12: Average training performances of SAGEConv on different distance cutoffs with 5-fold cross-validation. 28](#_Toc190271465)

[Table S13: Average training performances of SAGEConvMLP on different distance cutoffs with 5-fold cross-validation. 30](#_Toc190271466)

[Table S14: Average training performances of SAGEConvGATMLP on different distance cutoffs with 5-fold cross-validation. 32](#_Toc190271467)

[Validation Performances 34](#_Toc190271468)

[Without DSSP features 34](#_Toc190271469)

[Table S15: Validation performances of GCNConv on different distance cutoffs for DevSet1010. 34](#_Toc190271470)

[Table S16: Validation performances of SAGEConv on different distance cutoffs for DevSet1010. 36](#_Toc190271471)

[Table S17: Validation performances of SAGEConvMLP on different distance cutoffs for DevSet1010. 38](#_Toc190271472)

[Table S18: Validation performances of SAGEConvGATMLP on different distance cutoffs for DevSet1010. 40](#_Toc190271473)

[Table S19: Embedding comparison on SAGEConv model (cutoff: 17Å) for DevSet1010. 42](#_Toc190271474)

[With DSSP features 43](#_Toc190271475)

[Table S20: Validation performances of GCNConv on different distance cutoffs for DevSet1010. 43](#_Toc190271476)

[Table S21: Validation performances of SAGEConv on different distance cutoffs for DevSet1010. 45](#_Toc190271477)

[Table S22: Validation performances of SAGEConvMLP on different distance cutoffs for DevSet1010. 47](#_Toc190271478)

[Table S23: Validation performances of SAGEConvGATMLP on different distance cutoffs for DevSet1010. 49](#_Toc190271479)

[References for Supporting Online Material 51](#_Toc190271480)

# Short Description of Supporting Online Material

In this document, we provide additional tables and figures that support and expand upon our main work. We included detailed information about the used datasets. Additional performance comparisons and results from hyperparameter optimization experiments.

First, we include a table with the protein identifiers from the original DevSet1014, for which 3D structure prediction was not possible (Table S1). Additionally, we provide a table with the per-residue embedding dimensions for the pLM embeddings investigated in this work (Table S2) and a table on the class distribution in our training and test set (Table S3). Furthermore, we list Methods excluded for comparison (Table S4).

Next, we present Additional Results. In this section, we mainly provide additional insights on our comparison to bindEmbed21DL^1^. Table S5 contains additional performance measures for comparing bindNode24, bindEmbed21DL^1^, and GRaSP^2^. Figure S1 provides a scatter plot comparing per-residue predictions of bindNode24, bindEmbed21DL^1^, and Figure S2 heatmaps visualizing the differences of the prediction outputs. The reliability of both methods is further assessed through heatmaps in Figure S3 and a line plot (Figure S4). To further evaluate model performance, we included Receiver Operating Characteristic (ROC) and Precision-Recall (PR) curves (Figure S5, S6). Additionally, we investigated the relationship between plDDT confidence scores and predictions from bindNode24 (Figure S7).

Finally, we present results from our hyperparameter optimization, exploring the effect of different model architectures, input types, and structural cutoffs (Table S6-S23). The performance tables are separated into training (Table S6-S14) and validation performance (Table S15-S23). Within each set of tables, we distinguish between models that incorporate DSSP features (Tables S11-S14, S20-S23) and those that do not (Tables S6-S10, S15-S19). Additionally, we investigate the impact of different embedding choices at the optimal structure cutoff for our best-performing model SAGEConv without DSSP features (Table S10, S19).

# Materials and Methods

Table S1: Proteins that did not yield predicted structures.

| **Protein ID** | **Missing in** |
| --- | --- |
| **Q00277** | DevSet |
| **P84801** | DevSet |
| **Q9NZV6** | DevSet |
| **C8BD48** | DevSet |

Listed proteins have been removed from training due to ColabFold2 yielding no structure predictions. All compared methods have been trained on the subset without these proteins to ensure a fair comparison between different methods.

Table S2: Per-residue embedding dimensions.

| pLM | Vector Size |
| --- | --- |
| ProtT5 | 1024 |
| ProstT5 | 1024 |
| Ankh base | 768 |
| Ankh large | 1536 |
| ESM-2 (3B) | 2560 |
| ProtBert | 1024 |
| DistilProtBert | 1024 |
| OntoProtein | 1024 |

Embedding dimensions varied depending on the used pLM. The dimensions of all evaluated embedding types are shown. Embedding dimensions deviating from 1024 required adjustments of the input dimension of models trained with the corresponding embedding type.

Table S3: Dataset distributions.

|  |  | DevSet1010 | TestSet300 |
| --- | --- | --- | --- |
| All | # Proteins | 1,010 | 300 |
|  | # Residues | 170,181 | 62,689 |
|  | # Binding residues | 13,950 | 5,869 |
|  | # Non-binding residues | 156,231 | 56,820 |
| Metal | # Proteins | 454 | 124 |
|  | # Residues | 79,662 | 27,644 |
|  | # Binding residues | 2,370 | 881 |
|  | # Non-binding residues | 77,272 | 26,763 |
| Nuclear | # Proteins | 108 | 66 |
|  | # Residues | 18,271 | 16,168 |
|  | # Binding residues | 2,689 | 1,470 |
|  | # Non-binding residues | 15,582 | 14,698 |
| Small | # Proteins | 603 | 220 |
|  | # Residues | 103,011 | 46,535 |
|  | # Binding residues | 9,236 | 3,906 |
|  | # Non-binding residues | 93,775 | 42,629 |

Number of proteins binding and non-binding residues for all four binding prediction tasks (binary binding/non-binding, metal binding, nuclear binding, small binding) for training (DevSet1010) and test set (TestSet300). Values do not sum up to the numbers in the “all” row due to some residues being annotated with multiple ligand binding classes. Residue numbers always only consider residues in protein sequences, with at least one occurrence of this ligand being annotated in the sequence.

Pseudocode snippet: DSSP feature normalization.

**from** sklearn **import** preprocessing

**def** normalize_dssp_features**(**per_protein_dssp_features**):**

positions, dssp_structures, solvent_accessibilities,

phi_angles, psi_angles, NH_O_1_relative_index,

NH_O_1_relative_energy, O_NH_1_relative_index,

O_NH_1_relative_energy, NH_O_2_relative_index,

NH_O_2_relative_energy, O_NH_2_relative_index,

O_NH_2_relative_energy = per_protein_dssp_features

min_max_scaler **=** preprocessing**.**MinMaxScaler**()**

sequence_length **=** **len(**positions**)**

# convert absolute position in sequence to relative position

relative_index **=** [pos/sequence_length **for** pos **in** positions]

# one-hot encode secondary structures

secondary_structure_one_hot_encoding **=**

**[**one_hot_encode_dssp_structure**(**entry**)** **for** entry **in**

dssp_structures**]**

# keep solvant accesibility as it is (already normalized)

normalized_solvent_accessibility **=** solvent_accessibilities

# phi and psi angles are degrees => division by 360

normalized_phi **=** [angle/360 **for** angle **in** phi_angles]

normalized_psi **=** [angle/360 **for** angle **in** psi_angles]

# use normalization from sci-kit to normalize relative index

# and energies per protein

normalized_NH_O_1_relative_index **=**

min_max_scaler**.**fit_transform**(**NH_O_1_relative_index**)**

normalized_NH_O_1_relative_energy **=**

min_max_scaler**.**fit_transform**(**NH_O_1_relative_energy**)**

normalized_O_NH_1_relative_index **=**

min_max_scaler**.**fit_transform**(**O_NH_1_relative_index**)**

normalized_O_NH_1_relative_energy **=**

min_max_scaler**.**fit_transform**(**O_NH_1_relative_energy**)**

normalized_NH_O_2_relative_index **=**

min_max_scaler**.**fit_transform**(**NH_O_2_relative_index**)**

normalized_NH_O_2_relative_energy **=**

min_max_scaler**.**fit_transform**(**NH_O_2_relative_energy**)**

normalized_O_NH_2_relative_index **=**

min_max_scaler**.**fit_transform**(**O_NH_2_relative_index**)**

normalized_O_NH_2_relative_energy **=**

min_max_scaler**.**fit_transform**(**O_NH_2_relative_energy**)**

normalized_features **=** **[**

relative_index**,**

secondary_structure_one_hot_encoding**,**

normalized_solvent_accessibility**,**

normalized_phi**,**

normalized_psi**,**

normalized_NH_O_1_relative_index**,**

normalized_NH_O_1_relative_energy**,**

normalized_O_NH_1_relative_index**,**

normalized_O_NH_1_relative_energy**,**

normalized_NH_O_2_relative_index**,**

normalized_NH_O_2_relative_energy**,**

normalized_O_NH_2_relative_index**,**

normalized_O_NH_2_relative_energy

**]**

**return** normalized_features

Pseudocode snippet of the DSSP feature normalization procedure. Object conversions in the original code have been omitted to improve readability. As the first step, the sequence length of the protein is determined. Afterwards, features are normalized individually.

Sequence positions are converted to relative positions between 0 and 1 by dividing each position by the protein length. DSSP secondary structures are converted into one-hot encodings. Solvent accessibility does not need to be processed further because it is already provided normalized. Phi and psi angles are provided in degrees and need to be divided by 360 to be normalized to be between 0 and 1. For all remaining relative indexes and energies, the scikit-learn MinMaxScalar^3^ is used to normalize them based on the minimum and maximum in the individual protein. All normalized features are combined into a per-protein list of normalized features and assigned to the corresponding graph node as features in a later processing step.

Additional performance measures: CovOneBind, True Positive Rate, False Positive Rate, and Pearson correlation.

In addition to the performance measures and evaluations provided in the main text, we evaluated several supplementary metrics in the Additional Results section to provide a more comprehensive model assessment. In addition to the already mentioned standard annotations from the main text, we used the following additional definitions: Positives (P) were all residues annotated as binding, and negatives (N) were all residues annotated as non-binding. Using these definitions, we calculated the following additional performance metrics:

$$True Positive Rate= \frac{TP}{P}$$

$$False Positive Rate= \frac{FP}{N}$$

Additionally, we included CovOneBind, which measures the fraction of proteins with binding annotations for which at least a single residue is predicted to be binding:

$$CovOneBind= \frac{Number of proteins with at least one binding residue predicted}{Number of proteins with binding annotations}$$

This performance measure was first introduced with bindEmbed21DL^1^ and can help experimentalists determine the likelihood of the absence of a prediction implying the absence of binding residues in a protein.

To further evaluate the relationship between structure prediction confidence and binding residue prediction, we computed the Pearson correlation coefficient (r) between the binding predictions of bindNode24 and the plDDT confidence scores of the predicted structures. The Pearson correlation coefficient quantifies the linear relationship between two continuous variables and is defined as:

$$r=\frac{\sum{(x}_{i}-\bar{x}){(y}_{i}-\bar{y})}{\sqrt{\sum{{(x}_{i}-\bar{x})}^{2}}\sqrt{\sum{{(y}_{i}-\bar{y})}^{2}}}$$

Where x_i_ and y_i_ represent individual data points from the two variables, and x̄ and ȳ are their respective means. A strong positive correlation (r close to 1) indicates that a high value of x tends to coincide with a high value of y. Similarly, a strongly negative correlation (r close to -1) indicates an inverse relationship between x and y. In contrast, a weak correlation (r close to 0) suggests there is no linear dependency between the two variables.

Table S4: Methods excluded for comparison and reason for exclusion

| **Exclusion  Reason**  **Methods** | The Web server does not allow batch processing | No Web server is available | The code is not available | Insufficient installation instructions | Installation was not possible | Insufficient instructions on data preprocessing | Prediction of protein-protein interaction site |
| --- | --- | --- | --- | --- | --- | --- | --- |
| COACH-D^4^ | X |  | X |  |  |  |  |
| DeepCSeqSite^5^ |  | X |  | X | X |  |  |
| DeepPPISP^6^ |  | X |  |  |  |  | X |
| DeepSite^7^ | X |  | X |  |  |  |  |
| DELIA^8^ | X |  | X |  |  |  |  |
| FTMap^9^ |  | X | X |  |  |  |  |
| GraphBind^10^ | X |  |  |  | X |  |  |
| GraphPLBR^11^ |  | X |  |  |  | X |  |
| LigBind^12^ | X |  |  |  | X |  |  |
| NABind^13^ | X |  |  | X | X |  |  |
| PGpocket^14^ |  | X |  |  | X |  |  |
| PPI-hotspot^ID15^ | X |  |  |  |  |  | X |
| PPINet^16^ |  | X |  |  |  |  | X |
| PUResNetV2.0^17^ | X |  |  |  | X |  |  |
| ScanNet^18^ | X |  |  |  |  |  | X |
| SCRIBER^19^ | X  (only up to 10 sequen-ces) |  |  |  |  |  | X |
| SiteRadar^20^ |  | X | X |  |  |  |  |

Many other methods focus on the prediction of binding residues. Many of those could not be compared to our new method. Here, we give an overview of the reasons for excluding them from our comparison.

# Additional Results

## Test Performances

Table S5: Comparison of bindEmbed21DL, bindNode24 and GRaSP Performances on TestSet300

|  | **Performance Measure** | **bindEmbed21DL** | **bindNode24** | **GRaSP** |
| --- | --- | --- | --- | --- |
| **Overall** | MCC | 0.41 ± 0.02 | 0.42 ± 0.03 | 0.37 ± 0.03 |
|  | F1 | 43 ± 2 | 44 ± 3 | 36 ± 3 |
|  | Precision | 46 ± 3 | 50 ± 3 | 57 ± 4 |
|  | Recall | 51 ± 3 | 51 ± 4 | 30 ± 3 |
|  | Accuracy | 89 ± 1 | 90 ± 1 | 91 ± 1 |
| **Metal** | MCC | 0.22 ± 0.04 | 0.26 ± 0.04 | - |
|  | F1 | 22 ± 4 | 26 ± 5 | - |
|  | Precision | 23 ± 4 | 30 ± 5 | - |
|  | Recall | 25 ± 5 | 27 ± 5 | - |
|  | Accuracy | 97.5 ± 0.4 | 97.7 ± 0.4 | - |
| **Nuclear** | MCC | 0.22 ± 0.06 | 0.25 ± 0.07 | - |
|  | F1 | 24 ± 6 | 26 ± 7 | - |
|  | Precision | 32 ± 7 | 35 ± 9 | - |
|  | Recall | 23 ± 6 | 28 ± 8 | - |
|  | Accuracy | 91 ± 2 | 90 ± 2 | - |
| **Small** | MCC | 0.31 ± 0.03 | 0.34 ± 0.03 | - |
|  | F1 | 33 ± 3 | 35 ± 3 | - |
|  | Precision | 34 ± 4 | 40 ± 4 | - |
|  | Recall | 37 ± 4 | 40 ± 4 | - |
|  | Accuracy | 91 ± 1 | 92 ± 1 | - |

MCC, F1, Precision, Recall and Accuracy for bindNode24, bindEmbed21DL^1^ and GRaSP^2^ on TestSet300. ± values mark 1.96% standard errors, i.e., the 95% confidence Interval (CI). Except for Accuracy for the nuclear binding residue prediction class, bindNode24 numerically (but not statistically significantly) outperforms bindEmbed21DL in every single performance measure. bindNode24 and bindEmbed21DL both outperform GRaSP. Due to GRaSP only predicting binary binding/non-binding, performances could only be compared to the overall performance.

Figure S1: Predicted probabilities of bindNode24 and bindEmbed21DL on TestSet300


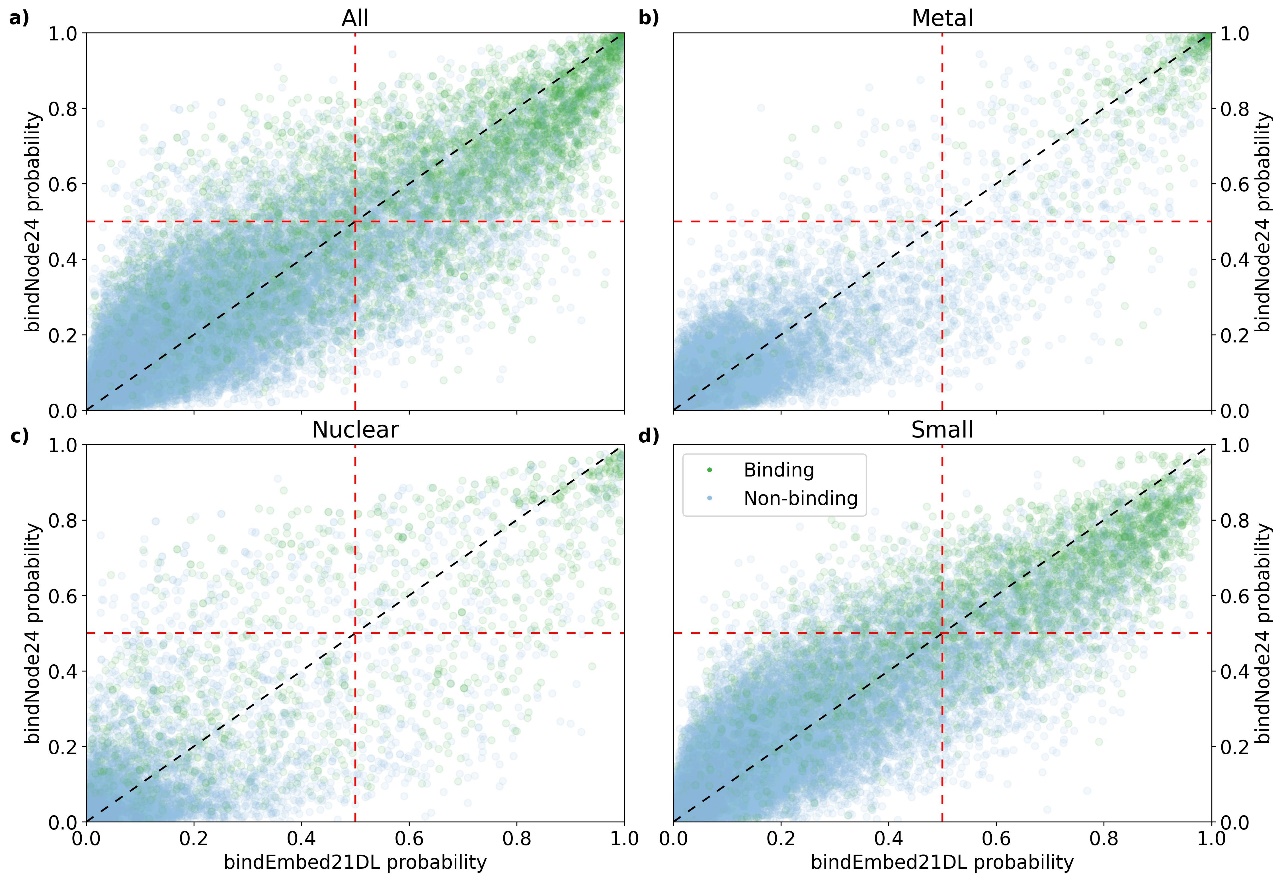


Predicted probabilities of bindNode24 vs. bindEmbed21DL: a) binary binding vs. non-binding, b) metal-binding, c) DNA/RNA-binding (nucleic acid-binding), d) small-molecule binding.

Residues annotated as binding are shown in green, and non-binding in blue. Red dashed lines mark the probability cutoff for bindNode24 and bindEmbed21DL to predict a residue as binding/non-binding (≥ 0.5 is predicted as binding). The diagonal black line indicates agreement in the probability prediction between both methods.

Figure S2: Heatmaps of bindNode24 and bindEmbed21DL predictions on TestSet300


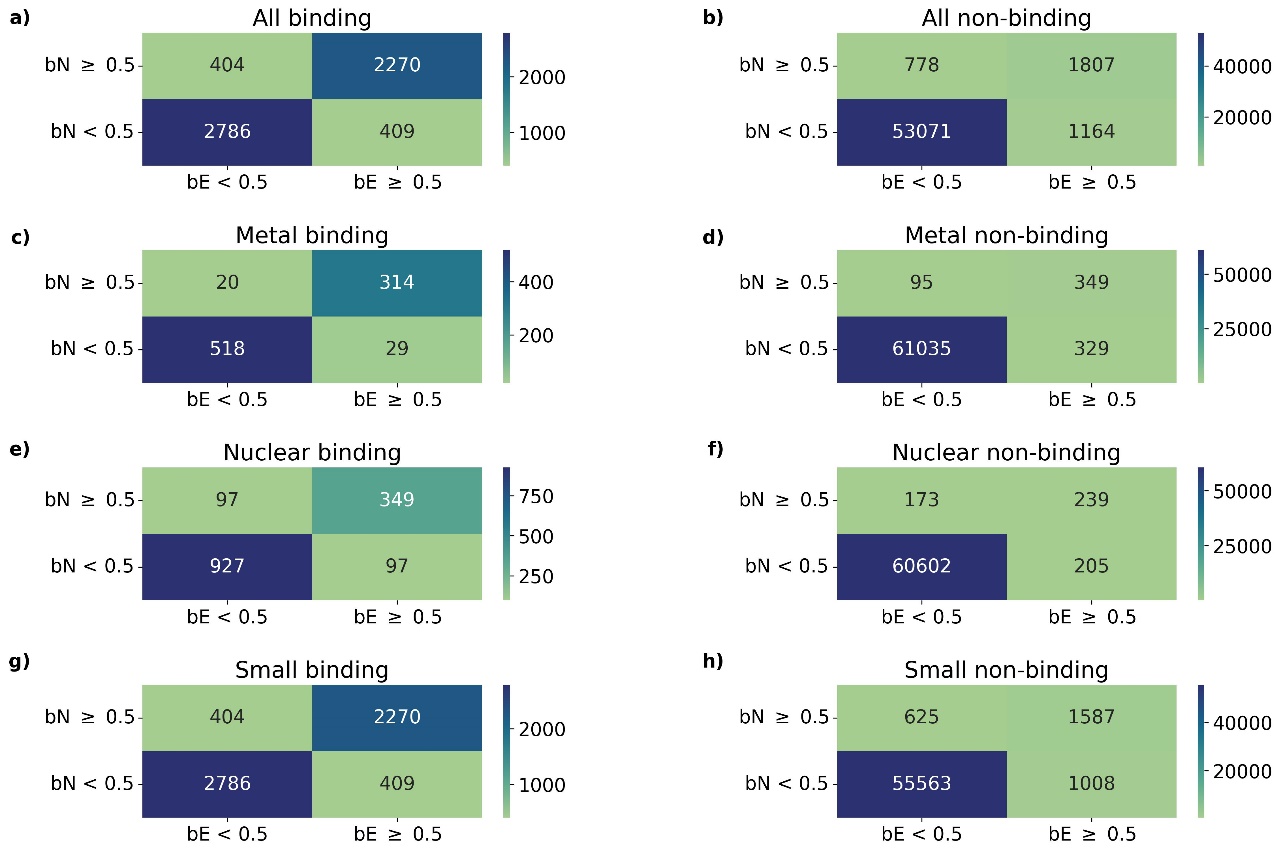


Heatmap comparison of bindNode24 and bindEmbed21DL predictions for: a & b) binary binding vs. non-binding, c & d) metal-binding, e & f) DNA/RNA-binding (nucleic acid-binding), g & h) small-molecule binding.

Residues are separated into annotated as binding (left column) and annotated as non-binding (right column).

In the left column, the top right entries show the number of residues for which both methods correctly predicted a residue as binding, and the bottom left entries show the number of residues that both methods failed to predict as binding. The top left entries show the number of residues that have been correctly predicted as binding by bindNode24 but missed by bindEmbed21DL, and the bottom right entries show the number correctly predicted by bindEmbed21DL but missed by bindNode24.

In the right column, the bottom left entries show the number of residues for which both methods correctly predicted a residue as non-binding, and the top right entries show the number of residues that both methods failed to predict as non-binding. The top left entries show the number of residues that have been correctly predicted as non-binding by bindEmbed21DL but missed by bindNode24, and the bottom right entries show the number correctly predicted by bindNode24 but missed by bindEmbed21DL.

While the numbers of residues correctly predicted as binding by one method but missed by the other are similar, bindNode24 is more successful at correctly predicting residues as non-binding that are incorrectly predicted to be binding by bindEmbed21DL than the other way around, explaining the consistent numerical advantage across most performance measures for bindNode24.

Abbreviations: bE: bindEmbed21DL; bN: bindNode24

Figure S3: Heatmaps of bindNode24 and bindEmbed21DL reliabilities on TestSet300


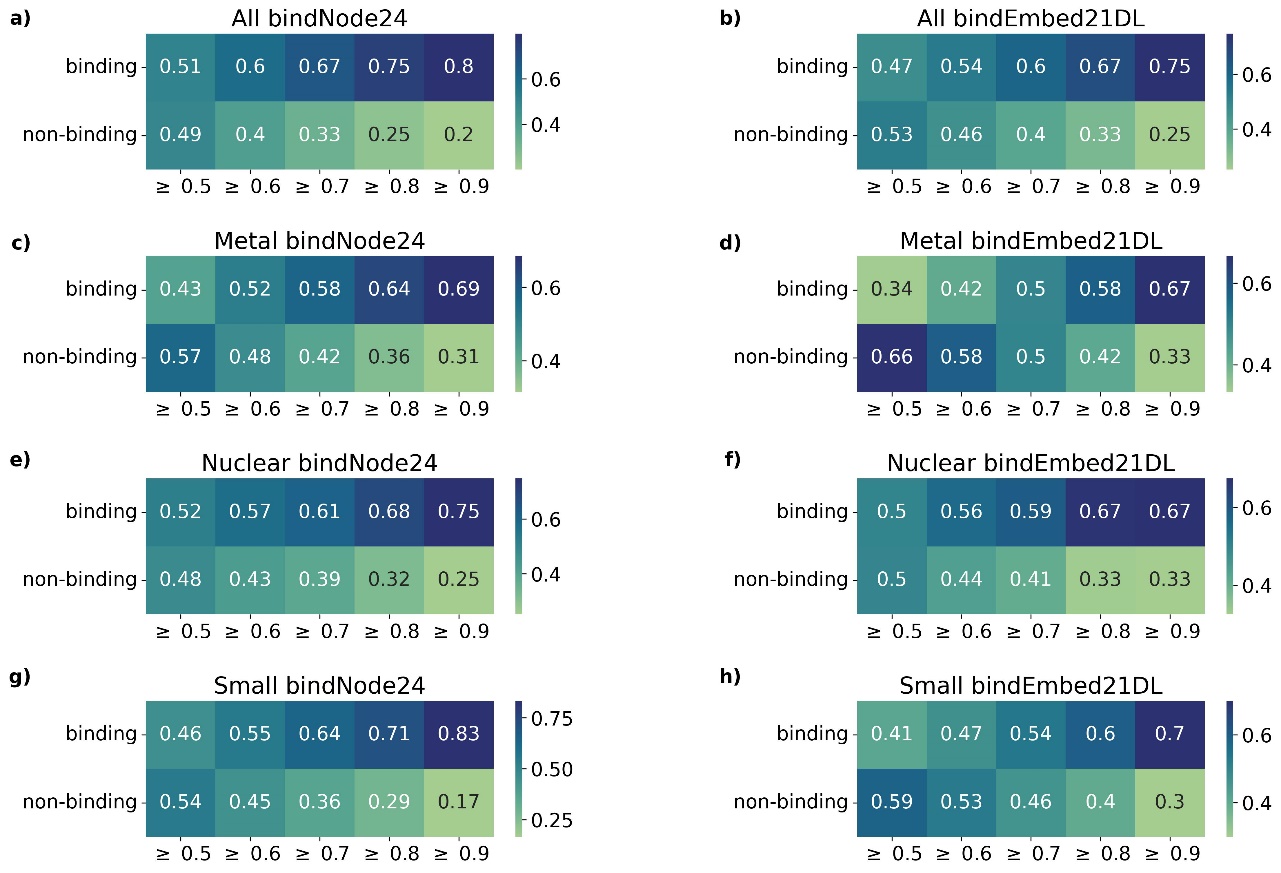


Heatmap comparison of bindNode24 and bindEmbed21DL reliabilities for: a & b) binary binding vs. non-binding, c & d) metal-binding, e & f) DNA/RNA-binding (nucleic acid-binding), g & h) small-molecule binding.

Each heatmap shows only the predictions made with a probability ≥ threshold t (0.5, 0.6, 0.7, 0.8, 0.9), which are then separated into two rows: residues correctly predicted as binding and residues incorrectly predicted as binding (i.e., non-binding residues that should not have exceeded the threshold). Higher thresholds correspond to more reliable predictions, as a smaller fraction of non-binding residues exceeds the threshold for both methods. bindNode24 consistently demonstrates a larger fraction of predictions above a given threshold t that are correctly identified as binding.

Figure S4: Precision, Recall and CovOneBind curves of bindNode24 and bindEmbed21DL on TestSet300


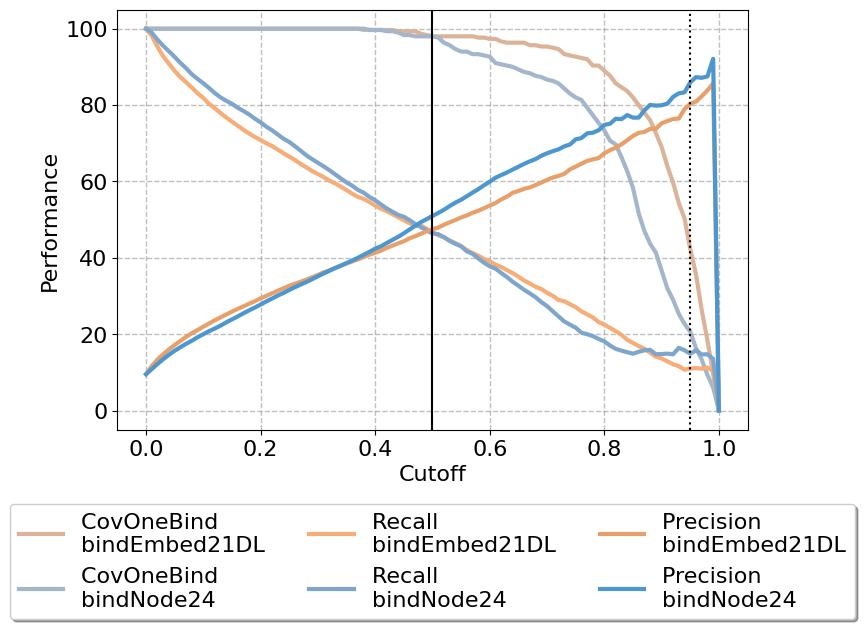


Higher predictions more often predict binding residues correctly. Precision and recall are computed for all proteins with at least one residue predicted as binding. The fraction of residues that is predicted to have at least a single residue predicted as binding is indicated by CovOneBind (see “Additional Performance Measure: CovOneBind”). The x-axis gives the output probability cutoff of bindEmbed21DL^1^ and bindNode24 for a prediction and the y-axis the performance. The solid black line marks the cutoff used in both models to consider a residue to be predicted as binding (≥ 0.5). Curves show the specific values for a chosen cutoff, e.g., the precision of all residues predicted with probability ≥ 0.95 (marked dashed line) was 87%, corresponding to a recall of 16% for bindNode24 (precision = 81%, recall = 11% for bindEmbed21DL). At this value, at least one binding residue was predicted for 16% (bindNode24) and 36% (bindEmbed21DL) of the proteins.

Dataset: TestSet300

Figure S5: Receiver Operating Characteristic (ROC) Curves of bindNode24 and bindEmbed21DL on TestSet300


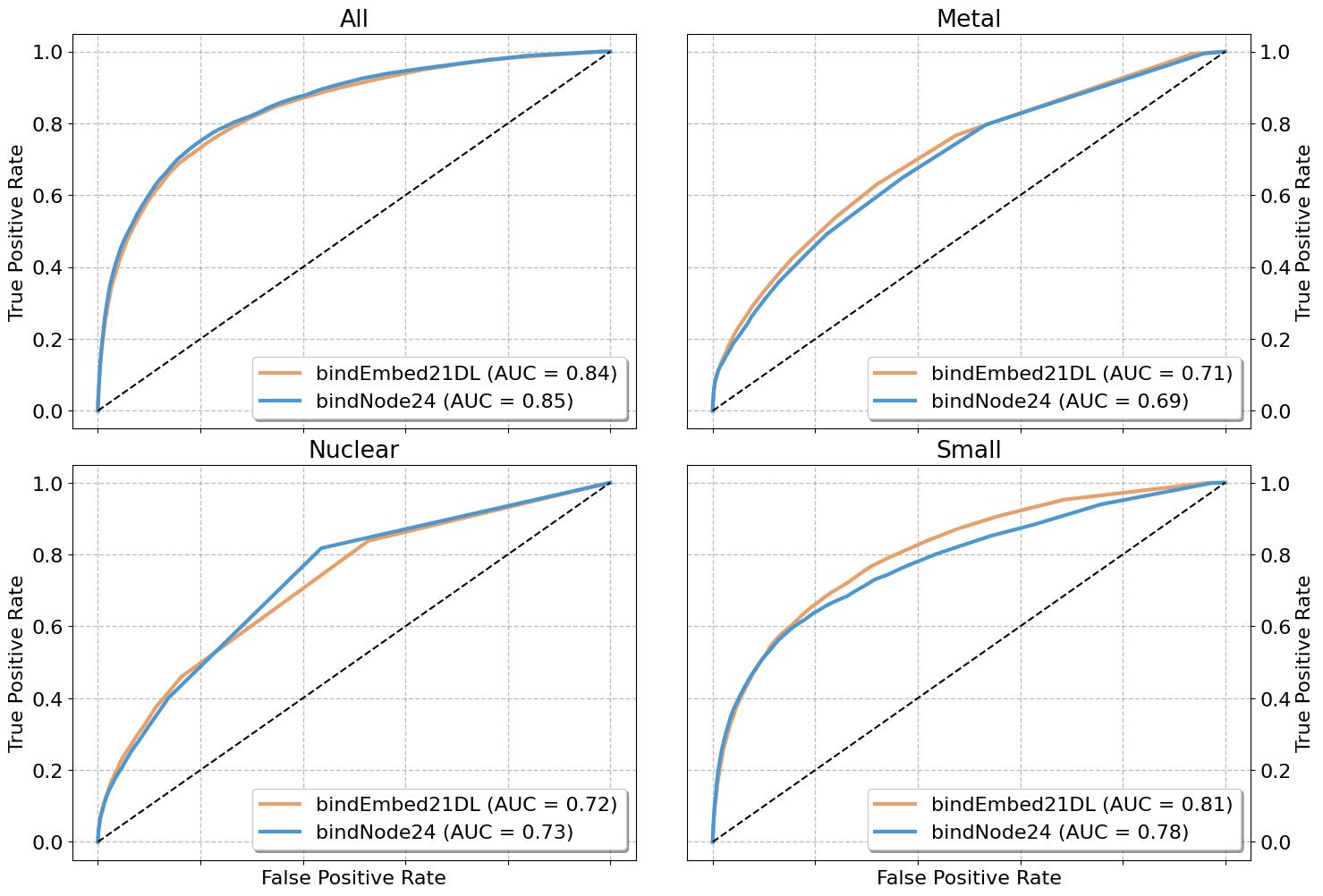


The ROC curve illustrates the trade-off between true positive rate and false positive rate for bindEmbed21DL^1^ and bindNode24. The area under the curve (AUC) indicates that bindEmbed21DL^1^ and bindNode24 discriminate similarly well for binding residue predictions, with bindNode24 achieving a slightly higher value on nucleic binding residues and bindEmbed21DL^1^ a minor advantage on small binding residues at certain cutoffs.

Dataset: TestSet300

Figure S6: Precision-Recall (PR) Curves of bindNode24 and bindEmbed21DL on TestSet300


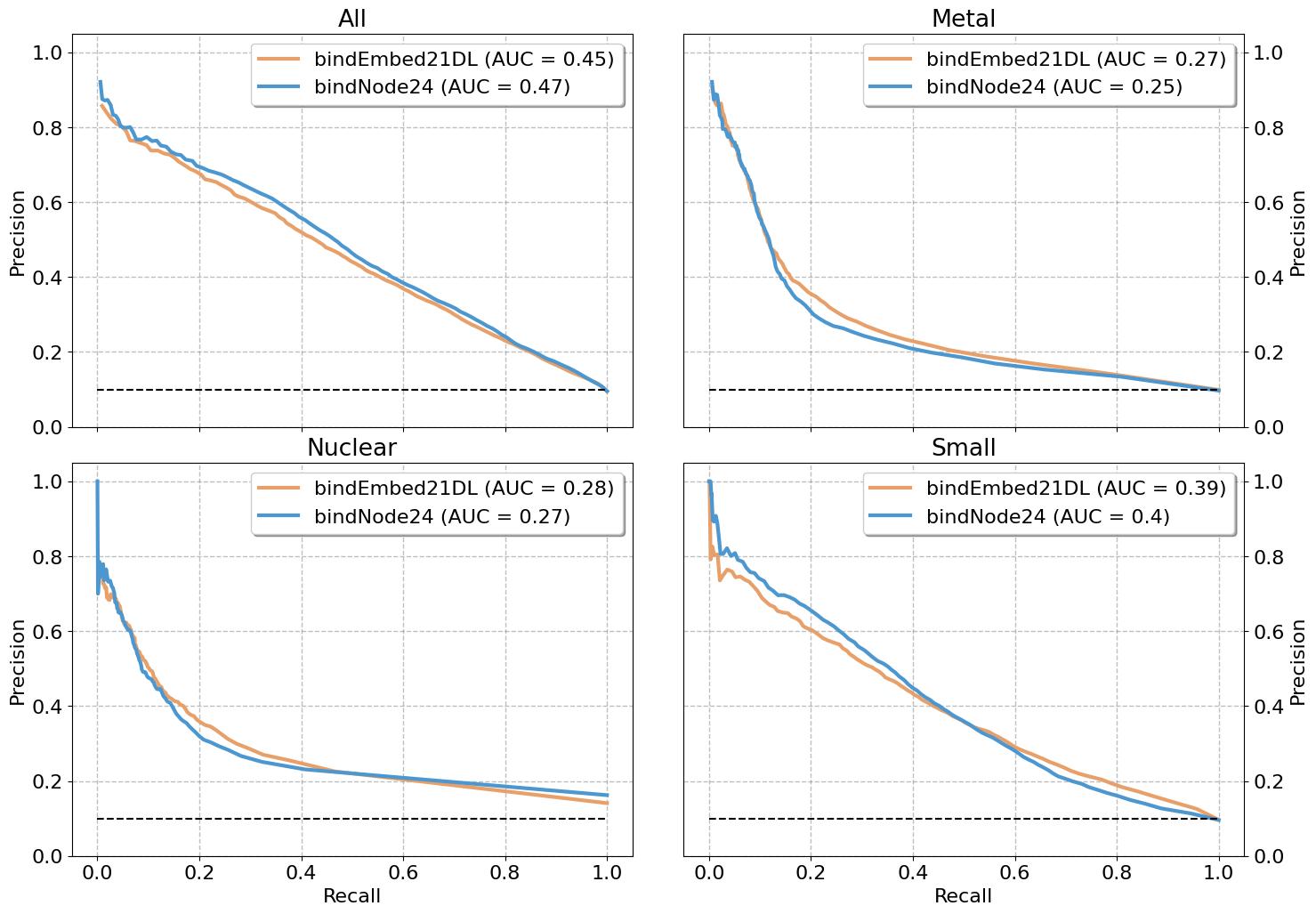


The PR curve illustrates the trade-off between precision and recall for bindEmbed21DL^1^ and bindNode24. The area under the curve (AUC) indicates that bindEmbed21DL^1^ and bindNode24 discriminate similarly well for binding residue predictions, with bindNode24 achieving a slightly higher value on the binary binding/non-binding task.

Dataset: TestSet300

Figure S7: Relationship between AlphaFold-predicted structure confidence (plDDT) and bindNode24 predictions


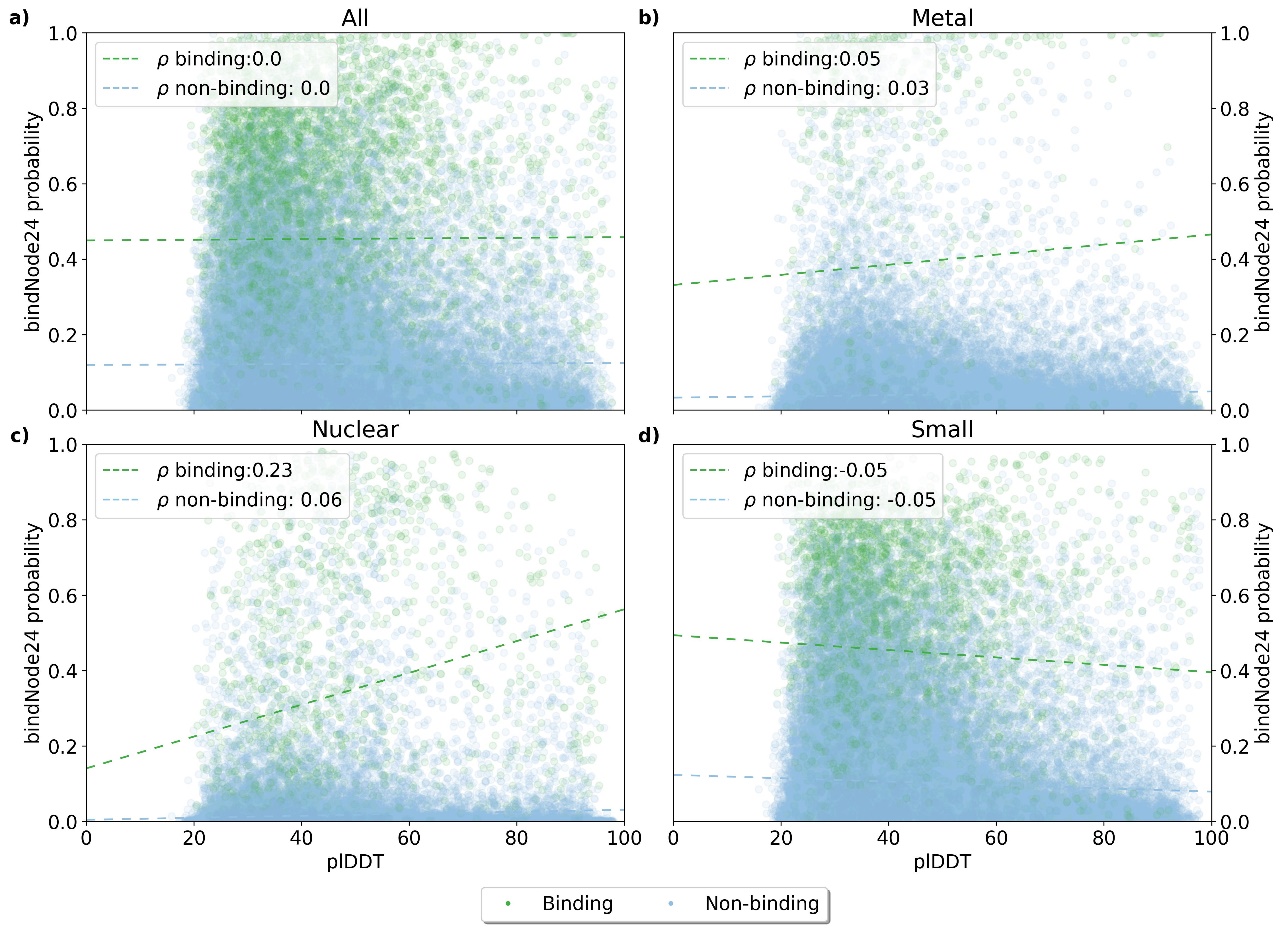


These scatterplots compare the per-residue plDDT-score from AlphaFold2^21^ (x-axis) against the bindNode24 predictions (y-axis). Color distinguishes the two annotations, binding and non-binding, used for assessment. Linear regression lines visualize the Pearson correlation for each annotation. Results for low Pearson correlation values (max 0.23 for binding residues of the nuclear class) indicate that AlphaFold’s plDDT hardly impacts our binding predictions (also indicated by the largely horizontal dashed lines). This suggests bindNode24 to be robust even in regions with lower structural confidence.

Dataset: TestSet300

## Training Performances

### Without DSSP features

Table S6: Average training performances of GCNConv on different distance cutoffs with 5-fold cross-validation.

| **Distance Cutoff** | **Overall** | | | | | **Metal** | | | | | **Nuclear** | | | | | **Small** | | | | |
| --- | --- | --- | --- | --- | --- | --- | --- | --- | --- | --- | --- | --- | --- | --- | --- | --- | --- | --- | --- | --- |
|  | MCC | F1 | Prec | Recall | Acc | MCC | F1 | Prec | Recall | Acc | MCC | F1 | Prec | Recall | Acc | MCC | F1 | Prec | Recall | Acc |
| **1** Å | 43 | **48** | 41 | 56 | **90** | 45 | **46** | **40** | 54 | **98** | 51 | 49 | 38 | 71 | **98** | 39 | 42 | 37 | 48 | **93** |
| **2** Å | 43 | **48** | **42** | 56 | **90** | **46** | **46** | **40** | 53 | **98** | 51 | 50 | 38 | 70 | **98** | 39 | 42 | **38** | 49 | **93** |
| **3** Å | **44** | **48** | **42** | 58 | **90** | **46** | **46** | 39 | **56** | **98** | 52 | 50 | 38 | 73 | **98** | **40** | **43** | **38** | **50** | **93** |
| **4** Å | 41 | 46 | 38 | **59** | 89 | 39 | 39 | 30 | 54 | **98** | **56** | **53** | **39** | **81** | **98** | 38 | 42 | 36 | **50** | 92 |
| **5** Å | 40 | 45 | 37 | 57 | 88 | 37 | 36 | 28 | 52 | 97 | 54 | 52 | 38 | 78 | **98** | 37 | 40 | 34 | 48 | 92 |
| **6** Å | 36 | 42 | 34 | 55 | 87 | 32 | 32 | 25 | 45 | 97 | 52 | 49 | 35 | 79 | 97 | 34 | 38 | 32 | 46 | 92 |
| **7** Å | 36 | 42 | 34 | 55 | 87 | 31 | 31 | 25 | 42 | 97 | 53 | 50 | 37 | 78 | **98** | 34 | 38 | 32 | 46 | 92 |
| **8** Å | 38 | 43 | 35 | 57 | 88 | 33 | 32 | 25 | 45 | 97 | 55 | 52 | 38 | **81** | **98** | 36 | 40 | 34 | 48 | 92 |
| **9** Å | 35 | 40 | 32 | 54 | 87 | 29 | 29 | 22 | 40 | 97 | 51 | 49 | 36 | 77 | 97 | 33 | 37 | 31 | 46 | 91 |
| **10** Å | 33 | 39 | 31 | 52 | 87 | 25 | 26 | 22 | 32 | 97 | 50 | 48 | 35 | 75 | 97 | 32 | 36 | 30 | 45 | 91 |
| **11** Å | 34 | 40 | 33 | 51 | 87 | 26 | 27 | 21 | 36 | 97 | 53 | 50 | 37 | 78 | **98** | 33 | 37 | 33 | 43 | 92 |
| **12** Å | 35 | 40 | 32 | 54 | 87 | 25 | 26 | 21 | 33 | 97 | 53 | 50 | 37 | 80 | 97 | 34 | 38 | 32 | 47 | 92 |
| **13** Å | 32 | 38 | 30 | 30 | 87 | 22 | 23 | 20 | 28 | 97 | 50 | 47 | 34 | 78 | 97 | 32 | 35 | 30 | 43 | 91 |
| **14** Å | 28 | 35 | 28 | 46 | 86 | 18 | 19 | 17 | 22 | 97 | 48 | 45 | 32 | 75 | 97 | 28 | 32 | 27 | 39 | 91 |
| **15** Å | 25 | 32 | 26 | 41 | 85 | 16 | 17 | 15 | 19 | 97 | 45 | 42 | 30 | 72 | 97 | 24 | 29 | 25 | 34 | 91 |
| **16** Å | 25 | 31 | 27 | 38 | 87 | 15 | 16 | 15 | 18 | 97 | 46 | 43 | 31 | 73 | 97 | 23 | 27 | 27 | 29 | 92 |
| **17** Å | 23 | 30 | 26 | 36 | 86 | 12 | 13 | 15 | 12 | **98** | 44 | 42 | 30 | 70 | 97 | 22 | 26 | 25 | 29 | 91 |
| **18** Å | 21 | 27 | 26 | 31 | 87 | 9 | 9 | 11 | 9 | **98** | 43 | 41 | 33 | 61 | 97 | 20 | 24 | 24 | 25 | 92 |
| **19** Å | 23 | 30 | 25 | 36 | 86 | 11 | 12 | 14 | 12 | **98** | 45 | 42 | 30 | 71 | 97 | 22 | 27 | 24 | 30 | 91 |
| **20** Å | 19 | 25 | 25 | 27 | 87 | 8 | 8 | 11 | 8 | **98** | 40 | 39 | 35 | 55 | 97 | 17 | 22 | 22 | 21 | 92 |

MCC, F1, Precision, Recall, and accuracy on the DevSet1010 for structure cutoffs from 1-20Å of the GCNConv model are shown and rounded to the nearest number. For all models, predicted AlphaFold 2^21^ structures were used. DSSP^22^ features were not used for the reported performances. The average performances of all five cross-validation training iterations on the four training splits are shown. Except for nuclear predictions, the model with only minimal structural information (3Å, approximately the distance between consecutive C_α_ atoms) achieves the best performance. An increase in the structure cutoff to include more distant amino acids leads to a drop in predictive performance. For predictions of nuclear binding residues, the best performance is achieved at a cutoff of 4Å. Similarly high performances for nuclear predictions are reached at a cutoff of 8 Å.

For each column, numerically highest performances are highlighted in bold.

Table S7: Average training performances of SAGEConv on different distance cutoffs with 5-fold cross-validation.

| **Distance Cutoff** | **Overall** | | | | | **Metal** | | | | | **Nuclear** | | | | | **Small** | | | | |
| --- | --- | --- | --- | --- | --- | --- | --- | --- | --- | --- | --- | --- | --- | --- | --- | --- | --- | --- | --- | --- |
|  | MCC | F1 | Prec | Recall | Acc | MCC | F1 | Prec | Recall | Acc | MCC | F1 | Prec | Recall | Acc | MCC | F1 | Prec | Recall | Acc |
| **1** Å | 42 | 47 | 41 | 55 | **90** | 44 | 45 | 38 | 53 | 98 | 49 | 48 | 36 | 70 | **98** | 37 | 40 | 36 | 47 | **93** |
| **2** Å | 43 | 48 | 41 | 57 | **90** | 46 | 47 | 42 | 54 | 98 | 51 | 50 | 38 | 72 | **98** | 39 | 42 | 36 | 50 | **93** |
| **3** Å | 42 | 47 | 41 | 55 | **90** | 44 | 45 | 39 | 53 | 98 | 50 | 48 | 37 | 70 | **98** | 37 | 41 | 36 | 47 | **93** |
| **4** Å | 43 | 48 | 41 | 58 | **90** | 45 | 45 | 39 | 53 | 98 | 53 | 51 | 39 | 74 | **98** | 39 | 42 | 37 | 51 | **92** |
| **5** Å | 44 | 48 | 41 | 60 | **90** | 47 | 47 | 44 | 51 | 98 | 54 | 52 | 41 | 73 | **98** | 40 | 43 | 36 | 54 | 92 |
| **6** Å | 46 | 50 | 42 | 61 | **90** | 48 | 48 | 43 | 55 | 98 | 56 | 54 | 41 | 79 | **98** | 42 | 45 | 39 | 54 | **93** |
| **7** Å | 46 | 50 | 43 | 60 | **90** | 46 | 47 | 40 | **56** | 98 | 56 | 54 | 40 | 80 | **98** | 42 | 45 | **40** | 52 | **93** |
| **8** Å | 44 | 49 | 42 | 59 | **90** | 46 | 47 | 43 | 51 | 98 | 55 | 53 | 41 | 75 | **98** | 41 | 44 | 38 | 53 | **93** |
| **9** Å | **47** | **51** | 43 | **63** | **90** | **49** | **50** | 46 | 55 | 98 | 57 | 55 | 41 | 82 | **98** | **44** | **46** | 39 | **57** | **93** |
| **10** Å | 45 | 49 | 42 | 61 | **90** | 47 | 47 | 44 | 53 | 98 | 55 | 53 | 40 | 78 | **98** | 42 | 45 | 38 | 54 | **93** |
| **11** Å | 45 | 50 | 43 | 60 | **90** | 48 | 49 | 46 | 52 | 98 | 56 | 54 | 42 | 77 | **98** | 42 | 45 | 38 | 55 | **93** |
| **12** Å | 45 | 50 | 42 | 62 | **90** | 47 | 48 | 45 | 52 | 98 | 56 | 54 | 41 | 78 | **98** | 42 | 45 | 38 | 56 | 92 |
| **13** Å | 46 | 50 | 42 | 61 | **90** | 47 | 47 | 42 | 53 | 98 | 56 | 53 | 40 | 81 | **98** | 42 | 45 | 39 | 54 | **93** |
| **14** Å | 46 | 50 | 42 | 62 | **90** | 47 | 48 | 43 | 54 | 98 | 56 | 54 | 41 | 80 | **98** | 43 | **46** | 38 | 56 | **93** |
| **15** Å | 45 | 50 | 42 | 60 | **90** | 47 | 48 | 47 | 50 | **99** | 55 | 53 | 41 | 78 | **98** | 42 | 45 | 38 | 55 | **93** |
| **16** Å | 46 | **51** | **44** | 60 | **90** | **49** | **50** | **49** | 50 | **99** | **58** | **56** | **43** | 80 | **98** | 43 | **46** | **40** | 54 | **93** |
| **17** Å | **47** | **51** | **44** | 62 | **90** | 48 | 49 | 44 | 54 | 98 | **58** | **56** | 42 | **83** | **98** | 43 | **46** | **40** | 56 | **93** |
| **18** Å | 45 | 49 | 42 | 60 | **90** | 47 | 48 | 46 | 50 | 98 | 56 | 53 | 41 | 79 | **98** | 41 | 44 | 38 | 54 | **93** |
| **19** Å | 46 | 50 | **44** | 59 | **90** | 47 | 48 | 45 | 51 | 98 | 57 | 54 | 41 | 81 | **98** | 42 | 45 | **40** | 52 | **93** |
| **20** Å | 46 | 50 | 42 | 62 | **90** | 47 | 47 | 44 | 52 | 98 | 56 | 54 | 40 | **83** | **98** | 42 | 45 | 38 | 55 | **93** |

MCC, F1, Precision, Recall, and accuracy on the DevSet1010 for structure cutoffs from 1-20Å of the SAGEConv model are shown, rounded to the nearest number. For all models, predicted AlphaFold 2^21^ structures were used. DSSP^22^ features were not used for the reported performances. The average performances of all five cross-validation training iterations are shown on the four training splits. Increasing the structure cutoff to include more distant amino acids generally improves performance. The best performance is achieved at 16-17Å.

For each column, numerically highest performances are highlighted in bold.

Table S8: Average training performances of SAGEConvMLP on different distance cutoffs with 5-fold cross-validation.

| **Distance Cutoff** | **Overall** | | | | | **Metal** | | | | | **Nuclear** | | | | | **Small** | | | | |
| --- | --- | --- | --- | --- | --- | --- | --- | --- | --- | --- | --- | --- | --- | --- | --- | --- | --- | --- | --- | --- |
|  | MCC | F1 | Prec | Recall | Acc | MCC | F1 | Prec | Recall | Acc | MCC | F1 | Prec | Recall | Acc | MCC | F1 | Prec | Recall | Acc |
| **1** Å | 31 | 37 | 32 | 45 | 87 | 20 | 17 | 11 | 48 | 93 | 21 | 18 | 11 | 50 | 93 | 24 | 28 | 22 | 39 | 89 |
| **2** Å | 30 | 36 | 30 | 47 | 87 | 19 | 16 | 10 | 50 | 92 | 19 | 16 | 9 | 52 | 91 | 23 | 27 | 21 | 38 | 89 |
| **3** Å | 29 | 35 | 28 | 47 | 86 | 17 | 14 | 8 | 50 | 91 | 18 | 14 | 8 | 51 | 90 | 23 | 27 | 21 | 39 | 89 |
| **4** Å | 30 | 36 | 31 | 46 | 87 | 19 | 16 | 10 | 47 | 93 | 20 | 17 | 11 | 50 | 92 | 24 | 28 | 22 | 40 | 89 |
| **5** Å | 31 | 37 | 35 | 41 | 89 | 20 | 18 | 12 | 46 | 94 | 25 | 23 | 16 | 51 | 94 | 24 | 28 | 26 | 33 | 91 |
| **6** Å | 32 | 38 | 35 | 43 | 88 | 20 | 18 | 12 | 45 | 94 | 27 | 24 | 16 | 53 | 94 | 24 | 28 | 24 | 36 | 90 |
| **7** Å | 31 | 36 | 31 | 46 | 86 | 20 | 17 | 11 | 46 | 92 | 22 | 20 | 13 | 48 | 93 | 23 | 27 | 21 | 39 | 88 |
| **8** Å | 32 | 38 | 34 | 43 | 88 | 19 | 17 | 10 | 45 | 93 | 24 | 21 | 14 | 50 | 94 | 24 | 28 | 24 | 36 | 90 |
| **9** Å | 34 | 39 | **41** | 39 | **90** | **23** | **23** | **16** | 40 | **96** | 30 | 29 | 20 | 52 | **96** | 25 | 29 | **28** | 32 | **92** |
| **10** Å | 31 | 37 | 33 | 45 | 88 | 19 | 17 | 10 | 46 | 93 | 25 | 22 | 15 | 54 | 93 | 24 | 28 | 23 | 37 | 89 |
| **11** Å | 33 | 39 | 38 | 41 | 89 | 21 | 19 | 12 | 46 | 94 | 29 | 26 | 18 | 55 | 95 | 24 | 28 | 26 | 32 | 91 |
| **12** Å | 33 | 38 | 34 | 45 | 88 | 19 | 16 | 10 | 47 | 93 | 27 | 23 | 15 | 59 | 94 | 24 | 28 | 23 | 37 | 90 |
| **13** Å | 34 | 39 | 38 | 41 | **90** | **23** | 22 | **16** | 41 | **96** | 32 | 30 | **22** | 53 | **96** | 26 | 30 | 27 | 34 | 91 |
| **14** Å | 32 | 37 | 33 | 45 | 88 | 19 | 16 | 10 | 47 | 93 | 26 | 22 | 14 | 56 | 93 | 23 | 27 | 22 | 36 | 90 |
| **15** Å | **35** | **40** | 36 | 49 | 88 | 21 | 19 | 13 | 45 | 93 | **33** | **31** | **22** | 61 | 95 | **28** | **31** | 27 | 41 | 90 |
| **16** Å | 34 | **40** | 38 | 42 | 89 | 21 | 19 | 12 | 42 | 95 | 32 | 29 | 20 | 59 | 95 | 25 | 29 | 27 | 33 | 91 |
| **17** Å | 34 | 39 | 38 | 42 | 89 | 20 | 18 | 12 | 43 | 94 | 31 | 28 | 19 | 58 | 95 | 26 | 30 | 27 | 34 | 91 |
| **18** Å | 27 | 33 | 26 | **55** | 77 | 15 | 12 | 7 | **53** | 83 | 21 | 17 | 10 | 61 | 88 | 22 | 26 | 20 | **43** | 84 |
| **19** Å | 33 | 39 | 37 | 42 | 89 | 20 | 17 | 11 | 43 | 94 | 28 | 25 | 17 | 53 | 95 | 25 | 29 | 25 | 35 | 90 |
| **20** Å | 32 | 37 | 32 | 47 | 87 | 18 | 15 | 9 | 47 | 92 | 25 | 20 | 13 | **62** | 91 | 24 | 29 | 24 | 36 | 90 |

MCC, F1, Precision, Recall, and accuracy on the DevSet1010 for structure cutoffs from 1-20Å of the SAGEConvMLP model are shown, rounded to the nearest number. For all models, predicted AlphaFold 2^21^ structures were used. DSSP^22^ features were not used for the reported performances. The average performances of all five cross-validation training iterations on the four training splits are shown. There is no clear best-performing cutoff; however, very high (>18Å) and very low (<5Å) cutoffs performed significantly worse in most performance measures.

For each column, numerically highest performances are highlighted in bold.

Table S9: Average training performances of SAGEConvGATMLP on different distance cutoffs with 5-fold cross-validation.

| **Distance Cutoff** | **Overall** | | | | | **Metal** | | | | | **Nuclear** | | | | | **Small** | | | | |
| --- | --- | --- | --- | --- | --- | --- | --- | --- | --- | --- | --- | --- | --- | --- | --- | --- | --- | --- | --- | --- |
|  | MCC | F1 | Prec | Recall | Acc | MCC | F1 | Prec | Recall | Acc | MCC | F1 | Prec | Recall | Acc | MCC | F1 | Prec | Recall | Acc |
| **1** Å | 45 | 49 | 38 | 68 | 88 | 47 | 45 | 34 | 68 | **98** | 54 | 51 | 38 | 82 | **98** | 40 | 42 | 33 | 58 | 91 |
| **2** Å | 45 | 49 | 38 | 68 | 88 | 47 | 45 | 34 | 68 | **98** | 54 | 51 | 38 | 82 | **98** | 40 | 42 | 33 | 58 | 91 |
| **3** Å | 47 | 51 | **39** | 71 | **89** | **52** | **49** | **37** | **75** | **98** | 56 | 53 | 39 | 84 | **98** | 42 | 44 | 34 | 62 | **92** |
| **4** Å | 48 | 51 | **39** | 73 | **89** | 48 | 46 | 35 | 68 | **98** | 61 | 57 | 42 | 90 | **98** | 43 | 45 | 34 | 64 | 91 |
| **5** Å | 29 | 36 | 27 | 68 | 70 | 29 | 28 | 21 | 51 | 90 | 37 | 37 | 27 | 67 | 86 | 26 | 31 | 23 | 55 | 79 |
| **6** Å | 27 | 35 | 26 | 66 | 70 | 27 | 27 | 20 | 51 | 87 | 37 | 35 | 26 | 64 | 89 | 24 | 29 | 21 | 51 | 79 |
| **7** Å | 37 | 42 | 32 | 69 | 79 | 35 | 34 | 26 | 57 | 93 | 48 | 46 | 33 | 80 | 92 | 33 | 37 | 28 | 58 | 85 |
| **8** Å | **49** | 51 | **39** | 76 | 88 | 47 | 45 | 34 | 68 | **98** | **63** | **59** | **44** | 92 | **98** | 45 | 46 | 35 | 69 | 91 |
| **9** Å | 43 | 46 | 36 | 66 | 88 | 41 | 40 | 31 | 58 | **98** | 53 | 50 | 38 | 77 | **98** | 39 | 41 | 32 | 60 | 91 |
| **10** Å | 33 | 38 | 29 | 63 | 79 | 30 | 30 | 23 | 49 | 92 | 39 | 37 | 27 | 64 | 93 | 30 | 34 | 26 | 53 | 86 |
| **11** Å | **49** | **52** | **39** | **77** | 88 | 46 | 44 | 34 | 66 | **98** | 62 | **59** | 43 | **93** | **98** | **47** | **48** | **36** | 71 | **92** |
| **12** Å | 39 | 44 | 32 | 73 | 79 | 35 | 35 | 26 | 56 | 94 | 50 | 47 | 35 | 80 | 91 | 37 | 39 | 29 | 63 | 86 |
| **13** Å | 48 | 51 | 38 | **77** | 88 | 45 | 43 | 32 | 67 | **98** | 62 | 58 | 43 | 91 | **98** | 46 | 47 | 35 | 71 | 91 |
| **14** Å | 48 | 50 | 38 | 76 | 88 | 44 | 43 | 33 | 64 | **98** | 61 | 57 | 42 | 92 | **98** | 45 | 46 | 35 | 70 | 91 |
| **15** Å | 48 | 51 | 38 | 76 | 88 | 45 | 43 | 32 | 65 | **98** | 61 | 57 | 42 | 91 | **98** | 46 | 47 | 35 | 71 | 91 |
| **16** Å | 48 | 51 | 38 | **77** | 88 | 46 | 45 | 34 | 66 | **98** | 61 | 57 | 42 | 92 | **98** | **47** | **48** | **36** | **72** | 91 |
| **17** Å | 47 | 50 | 38 | 75 | 88 | 45 | 43 | 33 | 64 | **98** | 59 | 55 | 40 | 91 | **98** | 46 | 47 | 35 | 70 | 91 |
| **18** Å | 47 | 49 | 37 | 74 | 88 | 43 | 42 | 31 | 63 | **98** | 59 | 56 | 40 | 90 | **98** | 45 | 46 | 35 | 69 | 91 |
| **19** Å | 47 | 50 | 37 | 74 | 88 | 44 | 42 | 32 | 64 | **98** | 59 | 55 | 40 | 90 | **98** | 45 | 46 | 35 | 68 | 91 |
| **20** Å | 48 | 50 | 38 | 76 | 88 | 45 | 43 | 32 | 66 | **98** | 61 | 57 | 42 | 91 | **98** | 46 | 47 | 35 | 71 | 91 |

MCC, F1, Precision, Recall, and accuracy on the DevSet1010 for structure cutoffs from 1-20Å of the SAGEConvGATMLP model are shown, rounded to the nearest number. For all models, predicted AlphaFold 2^21^ structures were used. DSSP^22^ features were not used for the reported performances. The average performances of all five cross-validation training iterations are shown on the four training splits. There is no clear single best model for all four prediction classes. The model at 11Å achieves the best performance in most evaluation metrics for the binary prediction task of binding vs. non-binding. For metal binding, the model with only minimal structure information (3Å) outperforms all other models for this prediction task. For the prediction of DNA and RNA binding residues, the model with the best trade-off overall evaluation metrics is the model trained with a structure cutoff at 8Å and for the predictions of binding residues for small molecules, both 11 Å and 16 Å show the most promising performance. Significantly lower performances for Models trained with a structure cutoff of 1-2Å show that models generally benefit from the integration of some structural information. However, no clear best structure cutoff for all four tasks can be determined, and some structure cutoffs (e.g., 6Å) lead to significantly lower prediction performances.

For each column, numerically highest performances are highlighted in bold.

Table S10: Embedding comparison on SAGEConv model (cutoff: 17Å) with 5-fold cross-validation.

| **Embedding** | **Overall** | | | | | **Metal** | | | | | **Nuclear** | | | | | **Small** | | | | |
| --- | --- | --- | --- | --- | --- | --- | --- | --- | --- | --- | --- | --- | --- | --- | --- | --- | --- | --- | --- | --- |
|  | MCC | F1 | Prec | Recall | Acc | MCC | F1 | Prec | Recall | Acc | MCC | F1 | Prec | Recall | Acc | MCC | F1 | Prec | Recall | Acc |
| **ProtT5** | **47** | **51** | 44 | **62** | 90 | **48** | **49** | **44** | **54** | **98** | **58** | **56** | 42 | **83** | **98** | **43** | **46** | 40 | **56** | **93** |
| **ProstT5** | 46 | **51** | **45** | 58 | **91** | 45 | 45 | 42 | 50 | **98** | **58** | **56** | 44 | 78 | **98** | **43** | **46** | **41** | 52 | **93** |
| **Ankh large** | 46 | 50 | 44 | 58 | **91** | 42 | 43 | 36 | 52 | **98** | 56 | **56** | **46** | 72 | **98** | 41 | 44 | 38 | 52 | **93** |
| **Ankh base** | 40 | 45 | 39 | 55 | 89 | 40 | 40 | 36 | 46 | **98** | 50 | 49 | 37 | 70 | **98** | 37 | 41 | 35 | 49 | 92 |
| **ESM-2 (3B)** | 42 | 47 | 42 | 55 | 90 | 43 | 43 | 39 | 49 | **98** | 56 | 53 | 40 | 80 | **98** | 38 | 42 | 38 | 47 | **93** |
| **ProtBert** | 38 | 43 | 38 | 50 | 89 | 39 | 40 | 36 | 44 | **98** | 52 | 59 | 35 | 78 | 97 | 34 | 37 | 35 | 41 | **93** |
| **DistilProtBert** | 34 | 39 | 36 | 44 | 89 | 35 | 36 | 33 | 40 | **98** | 46 | 44 | 33 | 69 | 97 | 29 | 33 | 32 | 34 | 92 |
| **OntoProtein** | 37 | 42 | 37 | 49 | 89 | 35 | 36 | 32 | 41 | **98** | 53 | 51 | 38 | 78 | **98** | 32 | 36 | 33 | 40 | 92 |

MCC, F1, Precision, Recall, and accuracy on the DevSet1010 for different protein language model embedding types (ProtT5^23^, ProstT5^24^, Ankh large^25^, Ankh base^25^, ESM^26^, ProtBert^23^, DistilProBert^27^ and OntoProtein^28^) for the SAGEConv model with a structure cutoff of 17Å are shown, rounded to the nearest number. For all models, predicted AlphaFold 2^21^ structures were used. DSSP^22^ features were not used for the reported performances

For each column, numerically highest performances are highlighted in bold.

### With DSSP features

Table S11: Average training performances of GCNConv on different distance cutoffs with 5-fold cross-validation.

| **Distance Cutoff** | **Overall** | | | | | **Metal** | | | | | **Nuclear** | | | | | **Small** | | | | |
| --- | --- | --- | --- | --- | --- | --- | --- | --- | --- | --- | --- | --- | --- | --- | --- | --- | --- | --- | --- | --- |
|  | MCC | F1 | Prec | Recall | Acc | MCC | F1 | Prec | Recall | Acc | MCC | F1 | Prec | Recall | Acc | MCC | F1 | Prec | Recall | Acc |
| **1** Å | 42 | 47 | **42** | 55 | **90** | 46 | **47** | **43** | 52 | **98** | 50 | 49 | 38 | 69 | **98** | 38 | 41 | 37 | 47 | **93** |
| **2** Å | **44** | **48** | **42** | **57** | **90** | 46 | **47** | 41 | **55** | **98** | 52 | **51** | **39** | 72 | **98** | **39** | **43** | **38** | **49** | **93** |
| **3** Å | **44** | **48** | **42** | **57** | **90** | **47** | **47** | 42 | 54 | **98** | 52 | **51** | **39** | 72 | **98** | **39** | **43** | **38** | **49** | **93** |
| **4** Å | 39 | 44 | 37 | **57** | 88 | 38 | 37 | 29 | 51 | **98** | **53** | **51** | 38 | 77 | **98** | 36 | 40 | 34 | 48 | 92 |
| **5** Å | 38 | 43 | 35 | **57** | 88 | 35 | 34 | 26 | 52 | 97 | 52 | 49 | 36 | 78 | 97 | 35 | 39 | 33 | 47 | 92 |
| **6** Å | 36 | 42 | 33 | 56 | 87 | 32 | 31 | 24 | 45 | 97 | 52 | 49 | 36 | 78 | 97 | 34 | 38 | 31 | 48 | 91 |
| **7** Å | 36 | 41 | 33 | 54 | 87 | 31 | 31 | 25 | 42 | 97 | 51 | 49 | 36 | 76 | 97 | 34 | 37 | 32 | 46 | 92 |
| **8** Å | 35 | 41 | 33 | 53 | 88 | 30 | 30 | 24 | 41 | 97 | 52 | 50 | **39** | 73 | **98** | 34 | 37 | 32 | 45 | 92 |
| **9** Å | 36 | 41 | 33 | 55 | 88 | 29 | 29 | 23 | 40 | 97 | 52 | 49 | 36 | **79** | 97 | 34 | 38 | 32 | 47 | 92 |
| **10** Å | 34 | 40 | 32 | 53 | 87 | 26 | 27 | 21 | 36 | 97 | 52 | 49 | 36 | 78 | 97 | 34 | 37 | 31 | 45 | 92 |
| **11** Å | 34 | 40 | 32 | 52 | 87 | 26 | 26 | 21 | 35 | 97 | 52 | 49 | 35 | **79** | 97 | 33 | 37 | 31 | 45 | 92 |
| **12** Å | 32 | 38 | 30 | 52 | 86 | 23 | 24 | 19 | 32 | 97 | 50 | 47 | 34 | 77 | 97 | 32 | 35 | 29 | 45 | 91 |
| **13** Å | 33 | 38 | 31 | 51 | 87 | 23 | 23 | 19 | 31 | 97 | 52 | 50 | 37 | 78 | 97 | 32 | 36 | 30 | 44 | 91 |
| **14** Å | 30 | 36 | 29 | 47 | 86 | 19 | 20 | 17 | 24 | 97 | 49 | 46 | 33 | 77 | 97 | 30 | 34 | 29 | 40 | 91 |
| **15** Å | 28 | 35 | 28 | 45 | 86 | 17 | 18 | 17 | 21 | 97 | 49 | 46 | 33 | 76 | 97 | 28 | 32 | 27 | 39 | 91 |
| **16** Å | 27 | 33 | 28 | 41 | 86 | 16 | 18 | 16 | 20 | 97 | 48 | 45 | 31 | 79 | 97 | 26 | 30 | 28 | 33 | 92 |
| **17** Å | 25 | 31 | 27 | 38 | 86 | 14 | 14 | 15 | 15 | **98** | 47 | 43 | 30 | 75 | 97 | 24 | 28 | 26 | 31 | 92 |
| **18** Å | 24 | 31 | 26 | 37 | 86 | 12 | 13 | 14 | 13 | **98** | 46 | 43 | 30 | 75 | 97 | 23 | 27 | 26 | 29 | 92 |
| **19** Å | 22 | 29 | 25 | 35 | 86 | 10 | 11 | 14 | 10 | **98** | 45 | 41 | 28 | 77 | 96 | 21 | 26 | 25 | 27 | 92 |
| **20** Å | 21 | 27 | 26 | 30 | 87 | 8 | 8 | 14 | 7 | **98** | 43 | 40 | 28 | 70 | 97 | 19 | 23 | 24 | 22 | 92 |

MCC, F1, Precision, Recall, and accuracy on the DevSet1010 for structure cutoffs from 1-20Å of the GCNConv model are shown and rounded to the nearest number. For all models, predicted AlphaFold 2^21^ structures and DSSP^22^ features were used for the reported performances. The average performances of all five cross-validation training iterations on the four training splits are shown. Except for nuclear predictions, the model with only minimal structural information (3Å, approximately the distance between consecutive C_α_ atoms) achieves the best performance. An increase in the structure cutoff to include more distant amino acids leads to a drop in predictive performance. For predictions of nuclear binding residues, the best performance is achieved at a cutoff of 4Å.

For each column, numerically highest performances are highlighted in bold.

Table S12: Average training performances of SAGEConv on different distance cutoffs with 5-fold cross-validation.

| **Distance Cutoff** | **Overall** | | | | | **Metal** | | | | | **Nuclear** | | | | | **Small** | | | | |
| --- | --- | --- | --- | --- | --- | --- | --- | --- | --- | --- | --- | --- | --- | --- | --- | --- | --- | --- | --- | --- |
|  | MCC | F1 | Prec | Recall | Acc | MCC | F1 | Prec | Recall | Acc | MCC | F1 | Prec | Recall | Acc | MCC | F1 | Prec | Recall | Acc |
| **1** Å | 41 | 46 | 40 | 54 | **90** | 43 | 43 | 36 | 53 | 98 | 47 | 45 | 34 | 70 | 97 | 36 | 40 | 36 | 44 | **93** |
| **2** Å | 42 | 47 | 40 | 56 | **90** | 45 | 46 | 42 | 51 | 98 | 49 | 48 | 36 | 70 | **98** | 37 | 41 | 35 | 48 | 92 |
| **3** Å | 42 | 47 | 41 | 55 | **90** | 45 | 46 | 42 | 51 | 98 | 50 | 48 | 36 | 71 | **98** | 37 | 41 | 36 | 48 | **93** |
| **4** Å | 45 | 49 | 42 | 60 | **90** | 47 | 48 | 42 | **55** | 98 | 55 | 53 | 40 | 78 | **98** | 41 | 44 | 39 | 52 | **93** |
| **5** Å | 43 | 48 | 41 | 57 | **90** | 45 | 45 | 40 | 53 | 98 | 53 | 51 | 38 | 76 | **98** | 39 | 43 | 38 | 49 | **93** |
| **6** Å | 43 | 48 | 40 | 59 | 89 | 44 | 45 | 40 | 52 | 98 | 53 | 50 | 38 | 76 | **98** | 39 | 43 | 36 | 52 | 92 |
| **7** Å | 42 | 47 | 40 | 58 | 89 | 44 | 45 | 40 | 52 | 98 | 52 | 50 | 39 | 73 | **98** | 39 | 42 | 36 | 51 | 92 |
| **8** Å | 43 | 48 | 41 | 58 | **90** | 46 | 46 | 43 | 51 | 98 | 53 | 51 | 38 | 77 | **98** | 40 | 43 | 38 | 51 | **93** |
| **9** Å | 45 | 50 | 43 | 59 | **90** | 47 | 47 | 43 | 53 | 98 | 56 | 54 | 41 | 77 | **98** | 41 | 45 | 39 | 53 | **93** |
| **10** Å | **47** | **52** | **44** | 62 | **90** | **49** | **50** | 46 | **55** | 98 | **58** | **56** | **43** | 82 | **98** | **44** | **47** | **41** | 55 | **93** |
| **11** Å | 45 | 49 | 42 | 60 | **90** | 47 | 47 | 45 | 50 | 98 | 55 | 53 | 40 | 78 | **98** | 42 | 45 | 38 | 54 | **93** |
| **12** Å | 44 | 49 | 41 | 60 | **90** | 46 | 46 | 44 | 50 | 98 | 55 | 53 | 40 | 78 | **98** | 41 | 44 | 38 | 54 | **93** |
| **13** Å | 46 | 51 | 43 | 62 | **90** | 47 | 48 | 44 | 53 | 98 | **58** | 55 | 41 | 82 | **98** | 43 | 46 | 40 | 55 | **93** |
| **14** Å | 46 | 51 | 42 | **63** | **90** | 48 | 49 | 45 | 53 | 98 | **58** | 55 | 42 | **83** | **98** | 43 | 46 | 38 | **56** | **93** |
| **15** Å | 45 | 49 | 42 | 60 | **90** | 47 | 47 | 45 | 51 | 98 | 56 | 53 | 40 | 82 | **98** | 41 | 45 | 38 | 53 | **93** |
| **16** Å | 44 | 49 | 42 | 58 | **90** | 46 | 46 | 43 | 50 | 98 | 56 | 54 | 41 | 79 | **98** | 40 | 44 | 39 | 50 | **93** |
| **17** Å | 45 | 49 | 42 | 60 | **90** | 47 | 47 | **47** | 48 | **99** | 56 | 54 | 41 | 80 | **98** | 41 | 44 | 38 | 54 | **93** |
| **18** Å | 45 | 49 | 42 | 59 | **90** | 46 | 46 | 44 | 50 | 98 | 55 | 53 | 39 | 82 | **98** | 41 | 44 | 39 | 51 | **93** |
| **19** Å | 45 | 50 | 42 | 61 | **90** | 46 | 46 | 43 | 51 | 98 | 56 | 53 | 40 | **83** | **98** | 42 | 45 | 38 | 54 | **93** |
| **20** Å | 46 | 50 | 44 | 59 | **90** | 47 | 48 | 44 | 52 | 98 | 57 | 55 | 41 | **83** | **98** | 42 | 45 | 40 | 52 | **93** |

MCC, F1, Precision, Recall, and accuracy on the DevSet1010 for structure cutoffs from 1-20Å of the SAGEConv model are shown, rounded to the nearest number. For all models, predicted AlphaFold 2^21^ structures and DSSP^22^ features were used for the reported performances. The average performances of all five cross-validation training iterations are shown on the four training splits. Increasing the structure cutoff to include more distant amino acids generally improves binding residue prediction performance up to a cutoff of 10Å. Further increase of the structure cutoff beyond this value does not show significant improvements in performance anymore. The best performance is achieved at 10Å. However, these performances deviate only slightly from the ones at e.g. 14Å.

For each column, numerically highest performances are highlighted in bold.

Table S13: Average training performances of SAGEConvMLP on different distance cutoffs with 5-fold cross-validation.

| **Distance Cutoff** | **Overall** | | | | | **Metal** | | | | | **Nuclear** | | | | | **Small** | | | | |
| --- | --- | --- | --- | --- | --- | --- | --- | --- | --- | --- | --- | --- | --- | --- | --- | --- | --- | --- | --- | --- |
|  | MCC | F1 | Prec | Recall | Acc | MCC | F1 | Prec | Recall | Acc | MCC | F1 | Prec | Recall | Acc | MCC | F1 | Prec | Recall | Acc |
| **1** Å | 30 | 36 | 29 | 48 | 86 | 18 | 15 | 9 | **49** | 92 | 19 | 15 | 9 | 53 | 90 | 23 | 27 | 21 | 41 | 88 |
| **2** Å | 30 | 36 | 31 | 46 | 87 | 21 | 18 | 11 | 47 | 94 | 18 | 15 | 10 | 45 | 91 | 24 | 28 | 22 | 40 | 88 |
| **3** Å | 32 | 37 | 34 | 42 | 88 | 22 | 20 | 13 | 46 | 95 | 21 | 19 | 13 | 42 | 94 | 24 | 28 | 23 | 37 | 90 |
| **4** Å | 32 | 38 | 34 | 46 | 87 | 22 | 20 | 13 | 46 | 94 | 26 | 23 | 16 | 54 | 93 | 25 | 29 | 24 | 39 | 89 |
| **5** Å | 33 | 38 | 33 | 48 | 87 | 22 | 20 | 14 | 46 | 94 | 27 | 25 | 17 | 56 | 93 | 25 | 29 | 23 | 41 | 89 |
| **6** Å | 32 | 37 | 32 | **49** | 86 | 21 | 19 | 13 | 46 | 93 | 27 | 24 | 17 | 57 | 92 | 25 | 28 | 23 | **42** | 88 |
| **7** Å | 36 | 41 | 38 | 45 | 89 | 24 | 22 | 16 | 46 | 95 | 30 | 28 | 20 | 53 | 95 | 27 | 31 | 27 | 38 | 91 |
| **8** Å | 35 | 40 | 37 | 46 | 89 | 24 | 23 | 17 | 44 | 95 | 31 | 28 | 20 | 56 | 95 | 27 | 31 | 26 | 39 | 91 |
| **9** Å | 34 | 40 | 36 | 48 | 88 | 24 | 22 | 16 | 43 | 95 | 33 | 31 | 23 | 58 | 95 | 28 | 31 | 27 | 41 | 90 |
| **10** Å | 36 | 42 | 39 | 46 | 89 | 25 | 24 | 18 | 41 | **96** | 36 | 35 | 26 | 57 | 96 | 29 | 33 | 29 | 40 | 91 |
| **11** Å | 33 | 39 | 35 | 48 | 87 | 22 | 20 | 14 | 46 | 93 | 32 | 30 | 22 | 57 | 93 | 27 | 31 | 27 | 38 | 91 |
| **12** Å | 38 | 43 | 41 | 45 | 90 | 25 | 24 | 18 | 41 | 96 | 37 | 36 | 27 | 57 | 96 | 29 | 33 | 30 | 38 | **92** |
| **13** Å | 38 | 43 | 40 | 46 | 90 | **28** | **27** | **21** | 43 | **97** | 40 | 38 | 28 | **61** | 96 | 30 | 34 | 31 | 38 | **92** |
| **14** Å | 35 | 40 | 38 | 43 | 89 | 22 | 21 | 15 | 41 | 95 | 33 | 31 | 23 | 56 | 96 | 27 | 31 | 28 | 36 | 91 |
| **15** Å | **39** | **44** | **43** | 46 | **91** | 27 | 26 | 19 | 42 | **97** | **41** | **39** | **29** | **61** | **97** | **31** | **35** | **32** | 39 | **92** |
| **16** Å | 36 | 41 | 38 | 47 | 89 | 26 | 25 | 18 | 43 | 96 | 37 | 35 | 26 | 57 | 96 | 29 | 32 | 29 | 41 | 90 |
| **17** Å | 35 | 41 | 39 | 43 | 90 | 23 | 23 | 16 | 39 | 96 | 37 | 34 | 25 | **61** | 96 | 27 | 32 | 29 | 36 | **92** |
| **18** Å | 38 | **44** | 41 | 47 | 90 | 26 | 26 | 19 | 41 | 96 | 39 | 38 | 28 | **61** | 96 | 30 | 34 | 30 | 40 | 91 |
| **19** Å | ­35 | 41 | 38 | 44 | 89 | 22 | 21 | 15 | 40 | 95 | 33 | 32 | 23 | 55 | 96 | 27 | 31 | 27 | 37 | 91 |
| **20** Å | 38 | 43 | 42 | 45 | 90 | 24 | 23 | 17 | 39 | 96 | 38 | 36 | 26 | 59 | 96 | 30 | 34 | 30 | 38 | **92** |

MCC, F1, Precision, Recall, and accuracy on the DevSet1010 for structure cutoffs from 1-20Å of the SAGEConvMLP model are shown, rounded to the nearest number. For all models, predicted AlphaFold 2^21^ structures and DSSP^22^ features were used for the reported performances. The average performances of all five cross-validation training iterations on the four training splits are shown. Overall, the models at a structure cutoff of 15Å show the highest performances in many of the provided metrics for all classes. Only for the metal class, prediction performance at 13Å is higher. Models with no or minimal structural information (1-3Å) perform significantly worse.

For each column, numerically highest performances are highlighted in bold.

Table S14: Average training performances of SAGEConvGATMLP on different distance cutoffs with 5-fold cross-validation.

| **Distance Cutoff** | **Overall** | | | | | **Metal** | | | | | **Nuclear** | | | | | **Small** | | | | |
| --- | --- | --- | --- | --- | --- | --- | --- | --- | --- | --- | --- | --- | --- | --- | --- | --- | --- | --- | --- | --- |
|  | MCC | F1 | Prec | Recall | Acc | MCC | F1 | Prec | Recall | Acc | MCC | F1 | Prec | Recall | Acc | MCC | F1 | Prec | Recall | Acc |
| **1** Å | 13 | 22 | 19 | 42 | 69 | 9 | 11 | 9 | 25 | 86 | 7 | 8 | 8 | 15 | 91 | 10 | 16 | 14 | 31 | 77 |
| **2** Å | 13 | 22 | 19 | 42 | 69 | 9 | 11 | 9 | 25 | 86 | 7 | 8 | 8 | 15 | 91 | 10 | 16 | 14 | 31 | 77 |
| **3** Å | 16 | 24 | 23 | 35 | 78 | 12 | 13 | 11 | 21 | 92 | 9 | 11 | 11 | 14 | 94 | 12 | 18 | 17 | 27 | 83 |
| **4** Å | 19 | 27 | 25 | 38 | 78 | 16 | 17 | 15 | 26 | 92 | 13 | 14 | 16 | 16 | 94 | 15 | 20 | 19 | 30 | 83 |
| **5** Å | 17 | 25 | 25 | 43 | 72 | 17 | 18 | 17 | 33 | 86 | 15 | 16 | 21 | 23 | 87 | 14 | 20 | 19 | 29 | 83 |
| **6** Å | 19 | 25 | 25 | 26 | 88 | 16 | 17 | 15 | 20 | 97 | 12 | 13 | 14 | 12 | 97 | 14 | 19 | 19 | 19 | 91 |
| **7** Å | 19 | 27 | 25 | 38 | 78 | 16 | 17 | 15 | 26 | 92 | 12 | 13 | 15 | 15 | 94 | 15 | 20 | 19 | 30 | 83 |
| **8** Å | 21 | 28 | 28 | 28 | 88 | 19 | 20 | 18 | 24 | 97 | 15 | 16 | 17 | 16 | 97 | 17 | 21 | 22 | 21 | **92** |
| **9** Å | 26 | 32 | 35 | 30 | **90** | 25 | 26 | 25 | 28 | **98** | 24 | 24 | 30 | 21 | **98** | 21 | 25 | 27 | 24 | **92** |
| **10** Å | 22 | 29 | 28 | 38 | 80 | 20 | 20 | 20 | 30 | 94 | 20 | 21 | 22 | 30 | 91 | 18 | 23 | 22 | 27 | 88 |
| **11** Å | 27 | 33 | 35 | 32 | **90** | 28 | 28 | 27 | 31 | **98** | 25 | 25 | 34 | 21 | **98** | 23 | 26 | 27 | 27 | **92** |
| **12** Å | 28 | 34 | 36 | 32 | **90** | 29 | 30 | 27 | 35 | **98** | 26 | 26 | **36** | 21 | **98** | 23 | 27 | 28 | 27 | **92** |
| **13** Å | 35 | 40 | 36 | 46 | 89 | 34 | 34 | 28 | 46 | **98** | 38 | 37 | 34 | 47 | **98** | 30 | 34 | 30 | 39 | **92** |
| **14** Å | 33 | 39 | 34 | 45 | 89 | 31 | 31 | 25 | 43 | 97 | 35 | 35 | 30 | 46 | **98** | 28 | 32 | 28 | 38 | **92** |
| **15** Å | 36 | 40 | **37** | 48 | 89 | 36 | 35 | 28 | 50 | 97 | 40 | 38 | 33 | 54 | **98** | 30 | 33 | **31** | 39 | **92** |
| **16** Å | 31 | 37 | 35 | 40 | 89 | 30 | 30 | 26 | 40 | **98** | 33 | 34 | 33 | 37 | **98** | 26 | 30 | 28 | 33 | **92** |
| **17** Å | **39** | **44** | 36 | **58** | 88 | **37** | **36** | 27 | **54** | 97 | **47** | **45** | 33 | **70** | 97 | **34** | **38** | 30 | **49** | 91 |
| **18** Å | 36 | 41 | 36 | 50 | 89 | 35 | 35 | **29** | 48 | **98** | 42 | 40 | 34 | 57 | **98** | 32 | 35 | **31** | 43 | **92** |
| **19** Å | 38 | 43 | 34 | **58** | 88 | 36 | 35 | 27 | 53 | 97 | 45 | 42 | 31 | 69 | 97 | 33 | 36 | 29 | **49** | 91 |
| **20** Å | 34 | 39 | 36 | 47 | 89 | 33 | 33 | 27 | 44 | 97 | 39 | 38 | 32 | 54 | 97 | 29 | 33 | 30 | 39 | **92** |

FIX ME! MCC, F1, Precision, Recall, and Accuracy on the DevSet1010 for structure cutoffs from 1-20Å of the SAGEConvGATMLP model are shown, rounded to the nearest number. For all models, predicted AlphaFold 2^21^ structures and DSSP^22^ features were used for the reported performances. The average performances of all five cross-validation training iterations are shown on the four training splits. The models at a structure cutoff of 3Å show the highest performances in many of the provided metrics for all classes. Models using higher or lower structure cutoffs perform significantly worse.

For each column, numerically highest performances are highlighted in bold.

## Validation Performances

### Without DSSP features

Table S15: Validation performances of GCNConv on different distance cutoffs for DevSet1010.

| **Distance Cutoff** | **Overall** | | | | | **Metal** | | | | | **Nuclear** | | | | | **Small** | | | | |
| --- | --- | --- | --- | --- | --- | --- | --- | --- | --- | --- | --- | --- | --- | --- | --- | --- | --- | --- | --- | --- |
|  | MCC | F1 | Prec | Recall | Acc | MCC | F1 | Prec | Recall | Acc | MCC | F1 | Prec | Recall | Acc | MCC | F1 | Prec | Recall | Acc |
| **1** Å | 40 | **43** | **43** | 53 | **90** | 31 | 31 | 33 | 32 | **98** | 20 | 23 | 24 | 26 | **92** | 28 | 30 | **33** | 33 | **92** |
| **2** Å | 40 | **43** | **43** | 53 | **90** | **32** | 31 | **34** | 33 | **98** | 21 | 23 | 25 | 27 | **92** | **29** | 30 | **33** | 34 | **92** |
| **3** Å | **41** | **43** | **43** | **54** | **90** | **32** | **32** | **34** | 34 | **98** | 22 | 25 | 25 | 28 | **92** | **29** | **31** | **33** | 34 | **92** |
| **4** Å | 35 | 38 | 35 | 52 | 88 | 26 | 26 | 23 | **36** | 96 | **26** | 31 | 28 | 41 | 87 | 27 | 30 | 31 | **36** | 90 |
| **5** Å | 33 | 37 | 34 | 51 | 88 | 25 | 25 | 22 | 35 | 96 | 25 | 30 | 27 | 39 | 87 | 26 | 29 | 29 | 35 | 89 |
| **6** Å | 29 | 33 | 30 | 48 | 87 | 22 | 21 | 19 | 32 | 96 | 24 | 30 | 27 | 40 | 85 | 23 | 27 | 26 | 34 | 88 |
| **7** Å | 28 | 32 | 29 | 47 | 87 | 20 | 20 | 17 | 30 | 95 | 25 | 32 | **30** | 42 | 85 | 23 | 26 | 26 | 34 | 88 |
| **8** Å | 29 | 33 | 29 | 48 | 87 | 22 | 21 | 19 | 32 | 95 | **26** | 33 | 29 | 46 | 84 | 24 | 27 | 27 | **36** | 88 |
| **9** Å | 26 | 30 | 27 | 46 | 86 | 18 | 17 | 15 | 28 | 95 | 24 | 32 | 28 | 43 | 83 | 22 | 26 | 24 | 35 | 87 |
| **10** Å | 23 | 28 | 25 | 42 | 86 | 13 | 13 | 11 | 22 | 95 | 22 | 30 | 26 | 41 | 82 | 21 | 24 | 24 | 33 | 87 |
| **11** Å | 23 | 27 | 25 | 42 | 86 | 13 | 13 | 10 | 24 | 94 | 25 | 34 | **30** | 48 | 81 | 20 | 24 | 23 | 33 | 86 |
| **12** Å | 22 | 26 | 23 | 40 | 86 | 12 | 11 | 9 | 23 | 94 | 25 | **35** | 29 | **51** | 80 | 20 | 24 | 23 | 34 | 86 |
| **13** Å | 20 | 24 | 22 | 37 | 85 | 9 | 9 | 7 | 17 | 94 | 23 | 33 | 28 | 50 | 79 | 19 | 23 | 22 | 33 | 86 |
| **14** Å | 16 | 20 | 19 | 32 | 85 | 7 | 7 | 5 | 15 | 94 | 20 | 30 | 25 | 48 | 78 | 14 | 18 | 18 | 26 | 86 |
| **15** Å | 13 | 18 | 16 | 29 | 84 | 6 | 6 | 5 | 12 | 94 | 18 | 28 | 23 | 43 | 77 | 12 | 16 | 15 | 24 | 85 |
| **16** Å | 11 | 16 | 14 | 25 | 85 | 5 | 5 | 4 | 10 | 94 | 18 | 29 | 25 | 46 | 77 | 10 | 14 | 13 | 21 | 86 |
| **17** Å | 9 | 14 | 13 | 24 | 84 | 3 | 3 | 3 | 7 | 95 | 17 | 28 | 23 | 44 | 76 | 9 | 14 | 12 | 22 | 84 |
| **18** Å | 7 | 12 | 10 | 20 | 85 | 2 | 2 | 2 | 5 | 95 | 16 | 27 | 24 | 42 | 75 | 7 | 11 | 10 | 18 | 85 |
| **19** Å | 8 | 13 | 11 | 22 | 85 | 2 | 3 | 2 | 6 | 94 | 16 | 29 | 24 | 47 | 74 | 8 | 12 | 10 | 21 | 84 |
| **20** Å | 5 | 9 | 8 | 16 | 86 | 0 | 1 | 0 | 2 | 96 | 13 | 24 | 22 | 37 | 76 | 5 | 9 | 8 | 17 | 84 |

MCC, F1, Precision, Recall, and accuracy on the DevSet1010 for structure cutoffs from 1-20Å of the GCNConv model are shown and rounded to the nearest number. For all models, predicted AlphaFold 2^21^ structures were used. DSSP^22^ features were not used for the reported performances. The performances from predicting DevSet1010 and using the consensus prediction over all five models are shown. Except for nuclear predictions, the model with only minimal structural information (3Å, approximately the distance between consecutive C_α_ atoms) achieves the best performance. An increase in the structure cutoff to include more distant amino acids leads to a drop in predictive performance.

For each column, numerically highest performances are highlighted in bold.

Table S16: Validation performances of SAGEConv on different distance cutoffs for DevSet1010.

| **Distance Cutoff** | **Overall** | | | | | **Metal** | | | | | **Nuclear** | | | | | **Small** | | | | |
| --- | --- | --- | --- | --- | --- | --- | --- | --- | --- | --- | --- | --- | --- | --- | --- | --- | --- | --- | --- | --- |
|  | MCC | F1 | Prec | Recall | Acc | MCC | F1 | Prec | Recall | Acc | MCC | F1 | Prec | Recall | Acc | MCC | F1 | Prec | Recall | Acc |
| **1** Å | 40 | 42 | 43 | 53 | **90** | 31 | 31 | 33 | 32 | **98** | 21 | 24 | 25 | 27 | **92** | 28 | 30 | 33 | 34 | **91** |
| **2** Å | 41 | 44 | 43 | 55 | **90** | 33 | 32 | **35** | 34 | **98** | 23 | 26 | 27 | 28 | **92** | 29 | 31 | 33 | 35 | **91** |
| **3** Å | 40 | 43 | 42 | 53 | **90** | 31 | 31 | 34 | 32 | **98** | 22 | 25 | 26 | 27 | **92** | 28 | 30 | 32 | 34 | **91** |
| **4** Å | 40 | 43 | 43 | 54 | **90** | 32 | 31 | 33 | 33 | **98** | 24 | 28 | 27 | 33 | 90 | 30 | 32 | 34 | 36 | **91** |
| **5** Å | 41 | 43 | 42 | 55 | **90** | 32 | 31 | 33 | 33 | **98** | 25 | 29 | 28 | 34 | 90 | 29 | 32 | 33 | 37 | **91** |
| **6** Å | **42** | **45** | 43 | **56** | **90** | **34** | **33** | **35** | **36** | **98** | 28 | 32 | 30 | 38 | 90 | **31** | 33 | **35** | 39 | **91** |
| **7** Å | 41 | 44 | 42 | **56** | **90** | 32 | 31 | 33 | 34 | **98** | 26 | 31 | 29 | 38 | 89 | 30 | 33 | 34 | 39 | **91** |
| **8** Å | 41 | 44 | 43 | 54 | **90** | 32 | 31 | 33 | 34 | **98** | 26 | 31 | 29 | 37 | 89 | 30 | 32 | **35** | 37 | **91** |
| **9** Å | **42** | **45** | 43 | **56** | **90** | **34** | **33** | **35** | **36** | **98** | 30 | 34 | 33 | 42 | 89 | **31** | **34** | 34 | **41** | 90 |
| **10** Å | 40 | 43 | 42 | 54 | **90** | 32 | 32 | 33 | 34 | **98** | 27 | 32 | 30 | 40 | 88 | 30 | 32 | 34 | 38 | **91** |
| **11** Å | 41 | 44 | 43 | 54 | **90** | 32 | 32 | 34 | 34 | **98** | 29 | 34 | 33 | 41 | 88 | 30 | 32 | 33 | 38 | **91** |
| **12** Å | 41 | 44 | 43 | 55 | **90** | 32 | 32 | 34 | 34 | **98** | 29 | 34 | 33 | 43 | 88 | 30 | 32 | 33 | 39 | 90 |
| **13** Å | 41 | 43 | 43 | 54 | **90** | 32 | 31 | 33 | 34 | **98** | 29 | 34 | 32 | 43 | 88 | 30 | 33 | 34 | 38 | 90 |
| **14** Å | 41 | 44 | 44 | 55 | **90** | 32 | 32 | 34 | 34 | **98** | 30 | 35 | 32 | 44 | 87 | **31** | 33 | **35** | 39 | **91** |
| **15** Å | 40 | 43 | 43 | 54 | **90** | 33 | 32 | **35** | 33 | **98** | 30 | 35 | 33 | 43 | 88 | 30 | 32 | 33 | 38 | **91** |
| **16** Å | 41 | 44 | 44 | 54 | **90** | 32 | 32 | 34 | 33 | **98** | 32 | 37 | **36** | 46 | 88 | 30 | 32 | 34 | 38 | **91** |
| **17** Å | **42** | **45** | **45** | **56** | **90** | **34** | **33** | **35** | 35 | **98** | **33** | **38** | **36** | **48** | 88 | **31** | **34** | **35** | 39 | **91** |
| **18** Å | 40 | 43 | 43 | 54 | **90** | 31 | 31 | 33 | 33 | **98** | 30 | 35 | 33 | 44 | 88 | 29 | 31 | 34 | 37 | **91** |
| **19** Å | 40 | 43 | 43 | 54 | **90** | 32 | 32 | 34 | 33 | **98** | 32 | 37 | 34 | **48** | 87 | 30 | 32 | 34 | 38 | 90 |
| **20** Å | 41 | 44 | **45** | 54 | **90** | 33 | **33** | **35** | 34 | **98** | 30 | 36 | 32 | 46 | 87 | 30 | 33 | **35** | 37 | **91** |

MCC, F1, Precision, Recall, and accuracy on the DevSet1010 for structure cutoffs from 1-20Å of the SAGEConv model are shown, rounded to the nearest number. For all models, predicted AlphaFold 2^21^ structures were used. DSSP^22^ features were not used for the reported performances. The performances from predicting DevSet1010 and using the consensus prediction over all five models are shown. Increasing the structure cutoff to include more distant amino acids generally improves performance. The best performance is achieved at 17Å.

For each column, numerically highest performances are highlighted in bold.

Table S17: Validation performances of SAGEConvMLP on different distance cutoffs for DevSet1010.

| **Distance Cutoff** | **Overall** | | | | | **Metal** | | | | | **Nuclear** | | | | | **Small** | | | | |
| --- | --- | --- | --- | --- | --- | --- | --- | --- | --- | --- | --- | --- | --- | --- | --- | --- | --- | --- | --- | --- |
|  | MCC | F1 | Prec | Recall | Acc | MCC | F1 | Prec | Recall | Acc | MCC | F1 | Prec | Recall | Acc | MCC | F1 | Prec | Recall | Acc |
| **1** Å | 38 | 41 | 40 | 53 | 89 | 19 | 18 | 16 | **26** | 94 | 6 | 7 | 7 | 7 | 94 | 23 | 25 | 26 | **30** | 90 |
| **2** Å | 38 | 40 | 40 | 53 | 89 | 18 | 18 | 15 | **26** | 94 | 6 | 7 | 7 | 8 | 94 | 23 | 25 | 26 | 29 | 89 |
| **3** Å | 38 | 40 | 40 | 53 | 89 | 16 | 16 | 13 | **26** | 93 | 5 | 6 | 6 | 7 | 92 | 23 | 25 | 27 | 28 | 89 |
| **4** Å | 39 | 41 | 40 | 53 | 89 | 19 | 18 | 16 | **26** | 94 | 6 | 7 | 7 | 8 | 94 | 24 | 26 | 27 | 29 | 90 |
| **5** Å | 39 | 41 | **44** | 49 | **91** | 21 | 21 | 20 | **26** | 96 | 7 | 8 | 9 | 9 | **95** | 24 | 26 | 30 | 27 | **92** |
| **6** Å | 39 | 41 | 42 | 51 | 90 | 21 | 20 | 19 | **26** | 96 | 7 | 8 | 8 | 10 | **95** | 24 | 26 | 28 | 28 | 91 |
| **7** Å | 38 | 41 | 41 | 50 | 90 | 19 | 19 | 17 | **26** | 95 | 6 | 8 | 8 | 9 | **95** | 23 | 25 | 28 | 27 | 90 |
| **8** Å | 38 | 41 | 41 | 52 | 90 | 18 | 18 | 16 | **26** | 94 | 7 | 8 | 8 | 9 | **95** | 23 | 25 | 27 | 28 | 90 |
| **9** Å | 40 | 42 | 43 | 51 | 90 | **24** | **24** | **25** | 25 | **97** | 8 | **10** | 10 | 11 | **95** | 25 | 27 | 30 | 29 | **92** |
| **10** Å | 38 | 41 | 42 | 51 | 90 | 20 | 19 | 18 | **26** | 95 | 7 | 8 | 8 | 9 | **95** | 23 | 25 | 28 | 27 | 91 |
| **11** Å | 39 | 41 | 43 | 50 | 90 | 20 | 20 | 18 | **26** | 95 | 7 | 8 | 8 | 10 | **95** | 24 | 25 | 29 | 27 | **92** |
| **12** Å | 39 | 41 | 42 | 52 | 90 | 19 | 18 | 16 | **26** | 94 | 6 | 8 | 7 | 9 | 94 | 24 | 26 | 28 | 28 | 91 |
| **13** Å | 40 | 42 | **44** | 50 | **91** | **24** | **24** | **25** | **26** | **97** | 9 | 10 | 11 | 12 | **95** | **26** | **28** | **31** | **30** | **92** |
| **14** Å | 38 | 41 | 41 | 52 | 89 | 18 | 17 | 15 | **26** | 93 | 6 | 7 | 7 | 9 | 94 | 23 | 25 | 27 | 28 | 91 |
| **15** Å | **41** | **43** | **44** | 52 | **91** | 23 | 23 | 23 | **26** | 96 | **10** | **11** | **12** | **13** | **95** | 25 | 27 | 30 | 29 | **92** |
| **16** Å | 39 | 42 | 43 | 51 | 90 | 22 | 22 | 22 | 25 | 96 | 8 | 9 | 9 | 11 | **95** | 25 | 27 | 30 | 29 | **92** |
| **17** Å | 39 | 42 | 43 | 51 | 90 | 21 | 20 | 19 | **26** | 95 | 7 | 9 | 9 | 11 | 94 | 25 | 27 | 30 | 29 | **92** |
| **18** Å | 38 | 41 | 40 | **54** | 89 | 17 | 17 | 14 | **26** | 92 | 6 | 7 | 6 | 9 | 93 | 24 | 26 | 27 | **30** | 90 |
| **19** Å | 39 | 42 | 42 | 52 | 90 | 20 | 20 | 19 | 25 | 95 | 7 | 8 | 8 | 10 | 94 | 24 | 26 | 28 | 29 | 91 |
| **20** Å | 38 | 41 | 41 | 53 | 89 | 18 | 17 | 15 | **26** | 93 | 6 | 7 | 6 | 9 | 94 | 24 | 26 | 27 | 29 | 90 |

MCC, F1, Precision, Recall, and accuracy on the DevSet1010 for structure cutoffs from 1-20Å of the SAGEConvMLP model are shown, rounded to the nearest number. For all models, predicted AlphaFold 2^21^ structures were used. DSSP^22^ features were not used for the reported performances. The performances from predicting DevSet1010 and using the consensus prediction over all five models are shown. There is no single clear best-performing cutoff for all four prediction tasks; however, very high (>18Å) and very low (<5Å) cutoffs performed significantly worse in many performance measures.

For each column, numerically highest performances are highlighted in bold.

Table S18: Validation performances of SAGEConvGATMLP on different distance cutoffs for DevSet1010.

| **Distance Cutoff** | **Overall** | | | | | **Metal** | | | | | **Nuclear** | | | | | **Small** | | | | |
| --- | --- | --- | --- | --- | --- | --- | --- | --- | --- | --- | --- | --- | --- | --- | --- | --- | --- | --- | --- | --- |
|  | MCC | F1 | Prec | Recall | Acc | MCC | F1 | Prec | Recall | Acc | MCC | F1 | Prec | Recall | Acc | MCC | F1 | Prec | Recall | Acc |
| **1** Å | 43 | 46 | 41 | 62 | 89 | 36 | 35 | 38 | 37 | **98** | 21 | 23 | 21 | 30 | **92** | 29 | 31 | 31 | 38 | **91** |
| **2** Å | 43 | 46 | 41 | 62 | 89 | 36 | 35 | 38 | 37 | **98** | 21 | 23 | 21 | 30 | **92** | 29 | 31 | 31 | 38 | **91** |
| **3** Å | 44 | 47 | 44 | 60 | **90** | **40** | **39** | **44** | 39 | **98** | 24 | 27 | 26 | 33 | **92** | 30 | 32 | 33 | 38 | **91** |
| **4** Å | 47 | 49 | 44 | 66 | **90** | 36 | 36 | 38 | 38 | **98** | 35 | 39 | 33 | 51 | 90 | 33 | 35 | 34 | 43 | **91** |
| **5** Å | 44 | 47 | 41 | 66 | 89 | 34 | 34 | 35 | 37 | **98** | 31 | 34 | 29 | 47 | 89 | 31 | 33 | 32 | 42 | 90 |
| **6** Å | 43 | 45 | 39 | 66 | 88 | 30 | 30 | 30 | 35 | 97 | 36 | 40 | 34 | 55 | 88 | 30 | 32 | 29 | 42 | 89 |
| **7** Å | 44 | 47 | 40 | 68 | 88 | 31 | 31 | 30 | 36 | 97 | 40 | 45 | 37 | 61 | 88 | 31 | 33 | 30 | 44 | 89 |
| **8** Å | **48** | 50 | 44 | 68 | **90** | 37 | 37 | 37 | 41 | **98** | 44 | 50 | 40 | 70 | 87 | 34 | 37 | 34 | 46 | **91** |
| **9** Å | 47 | 50 | 44 | 66 | **90** | 37 | 37 | 37 | 41 | **98** | 45 | 51 | 42 | 69 | 87 | 34 | 36 | 34 | 45 | 90 |
| **10** Å | 45 | 48 | 42 | 67 | 89 | 34 | 33 | 34 | 37 | **98** | 46 | 52 | 45 | 68 | 87 | 32 | 35 | 31 | 46 | 90 |
| **11** Å | **48** | **51** | **45** | 67 | **90** | 36 | 36 | 36 | 40 | **98** | 51 | 58 | 46 | 80 | 86 | 35 | 38 | **35** | 48 | **91** |
| **12** Å | 46 | 49 | 43 | 68 | 89 | 33 | 33 | 32 | 39 | 97 | 49 | 55 | 47 | 73 | 87 | 34 | 37 | 33 | 47 | 90 |
| **13** Å | 47 | 50 | 43 | **70** | 89 | 36 | 36 | 35 | 41 | 97 | **54** | **61** | **51** | 80 | 86 | 34 | 37 | 32 | **50** | 90 |
| **14** Å | 47 | 50 | 43 | 67 | **90** | 35 | 35 | 34 | 40 | 97 | 50 | 57 | 47 | 77 | 86 | 34 | 37 | 33 | 48 | 90 |
| **15** Å | 47 | 50 | 44 | 65 | **90** | 35 | 34 | 34 | 40 | 97 | 50 | 56 | 46 | 76 | 86 | 35 | 38 | 34 | 47 | 90 |
| **16** Å | 47 | 50 | **45** | 64 | **90** | 36 | 36 | 35 | 41 | 97 | 52 | 59 | 50 | 79 | 85 | 34 | 38 | **35** | 47 | 90 |
| **17** Å | 46 | 49 | 43 | 66 | 89 | 35 | 35 | 34 | 40 | 97 | 50 | 57 | 47 | 79 | 84 | 34 | 37 | 33 | 47 | 90 |
| **18** Å | 45 | 49 | 42 | 66 | 89 | 34 | 34 | 33 | 39 | 97 | 48 | 56 | 44 | 80 | 84 | 33 | 36 | 32 | 48 | 90 |
| **19** Å | 45 | 48 | 42 | 64 | 89 | 33 | 33 | 32 | 38 | 97 | 49 | 57 | 48 | 76 | 85 | 33 | 36 | 33 | 45 | 90 |
| **20** Å | 47 | **51** | **45** | 65 | **90** | 37 | 37 | 36 | **43** | 97 | 53 | **61** | **51** | **81** | 85 | **36** | **39** | **35** | **50** | 90 |

MCC, F1, Precision, Recall, and accuracy on the DevSet1010 for structure cutoffs from 1-20Å of the SAGEConvGATMLP model are shown, rounded to the nearest number. For all models, predicted AlphaFold 2^21^ structures were used. DSSP^22^ features were not used for the reported performances. The performances from predicting DevSet1010 and using the consensus prediction over all five models are shown. There is no clear single best model for all four prediction classes. The model at 11Å achieves the best performance in most evaluation metrics for the binary prediction task of binding vs. non-binding. For metal binding, the model with only minimal structure information (3Å) outperforms all other models for this prediction task. For the prediction of DNA and RNA binding residues, the model with the best trade-off overall evaluation metrics is the model trained with a structure cutoff at 13Å and for the predictions of binding residues for small molecules, 20 Å shows the most promising performance. Significantly lower performances for Models trained with a structure cutoff of 1-2Å show that models generally benefit from the integration of some structural information. However, no clear best structure cutoff for all four tasks can be determined, and some structure cutoffs (e.g., 6Å) lead to significantly lower prediction performances.

For each column, numerically highest performances are highlighted in bold.

Table S19: Embedding comparison on SAGEConv model (cutoff: 17Å) for DevSet1010.

| **Embedding** | **Overall** | | | | | **Metal** | | | | | **Nuclear** | | | | | **Small** | | | | |
| --- | --- | --- | --- | --- | --- | --- | --- | --- | --- | --- | --- | --- | --- | --- | --- | --- | --- | --- | --- | --- |
|  | MCC | F1 | Prec | Recall | Acc | MCC | F1 | Prec | Recall | Acc | MCC | F1 | Prec | Recall | Acc | MCC | F1 | Prec | Recall | Acc |
| **ProtT5** | **42** | **45** | 45 | **56** | 90 | **34** | **33** | 35 | **35** | **98** | **33** | 38 | 36 | 48 | 88 | **31** | **34** | 35 | **39** | **91** |
| **ProstT5** | **42** | 44 | **47** | 51 | **91** | **34** | **33** | **38** | 33 | **98** | **33** | 37 | **38** | 43 | 89 | **31** | 33 | **37** | 37 | **91** |
| **Ankh large** | 41 | 44 | 43 | **56** | 90 | 30 | 30 | 31 | 33 | 97 | 28 | 32 | 35 | 37 | **90** | 30 | 32 | 32 | 37 | **91** |
| **Ankh base** | 37 | 40 | 40 | 51 | 89 | 29 | 29 | 31 | 30 | 97 | 21 | 26 | 25 | 33 | 87 | 27 | 29 | 32 | 35 | 90 |
| **ESM-2 (3B)** | 40 | 43 | 45 | 51 | 90 | 33 | 32 | 35 | 33 | **98** | **33** | **41** | 37 | **53** | 85 | 29 | 31 | 34 | 36 | 90 |
| **ProtBert** | 33 | 36 | 37 | 44 | 89 | 26 | 26 | 28 | 28 | 97 | 23 | 29 | 26 | 39 | 85 | 24 | 26 | 30 | 30 | 89 |
| **DistilProtBert** | 30 | 33 | 35 | 41 | 88 | 23 | 23 | 24 | 24 | 97 | 19 | 25 | 25 | 34 | 85 | 19 | 22 | 26 | 26 | 89 |
| **OntoProtein** | 32 | 35 | 38 | 42 | 89 | 25 | 25 | 27 | 27 | 97 | 27 | 34 | 32 | 47 | 84 | 22 | 24 | 31 | 27 | 89 |

MCC, F1, Precision, Recall, and accuracy on the DevSet1010 for different protein language model embedding types (ProtT5^23^, ProstT5^24^, Ankh large^25^, Ankh base^25^, ESM^26^, ProtBert^23^, DistilProtBert^27^ and OntoProtein^28^) for the SAGEConv model with a structure cutoff of 17Å are shown, rounded to the nearest number. The performances from predicting DevSet1010 and using the consensus prediction over all five models are shown. For all models, predicted AlphaFold 2^21^ structures were used. DSSP^22^ features were not used for the reported performances.
For each column, numerically highest performances are highlighted in bold.

### With DSSP features

Table S20: Validation performances of GCNConv on different distance cutoffs for DevSet1010.

| **Distance Cutoff** | **Overall** | | | | | **Metal** | | | | | **Nuclear** | | | | | **Small** | | | | |
| --- | --- | --- | --- | --- | --- | --- | --- | --- | --- | --- | --- | --- | --- | --- | --- | --- | --- | --- | --- | --- |
|  | MCC | F1 | Prec | Recall | Acc | MCC | F1 | Prec | Recall | Acc | MCC | F1 | Prec | Recall | Acc | MCC | F1 | Prec | Recall | Acc |
| **1** Å | 40 | **43** | 43 | 52 | **90** | **32** | **32** | **34** | 32 | **98** | 22 | 25 | 26 | 28 | **92** | 28 | 30 | 33 | 33 | **92** |
| **2** Å | **41** | **43** | **44** | 53 | **90** | **32** | **32** | **34** | 33 | **98** | 22 | 25 | 25 | 28 | **92** | **29** | **31** | **34** | 34 | **92** |
| **3** Å | **41** | **43** | 43 | **54** | **90** | 31 | 31 | 32 | 32 | **98** | 21 | 24 | 25 | 27 | **92** | **29** | **31** | **34** | 35 | **92** |
| **4** Å | 34 | 37 | 34 | 51 | 88 | 26 | 25 | 23 | 34 | 96 | **24** | 29 | 27 | 37 | 87 | 26 | 30 | 30 | **36** | 89 |
| **5** Å | 33 | 37 | 33 | 52 | 88 | 25 | 25 | 22 | **35** | 96 | **24** | 30 | 27 | 38 | 87 | 26 | 29 | 29 | 35 | 89 |
| **6** Å | 29 | 33 | 30 | 48 | 87 | 22 | 22 | 19 | 32 | 95 | 23 | 29 | 27 | 38 | 86 | 23 | 27 | 27 | 35 | 88 |
| **7** Å | 28 | 32 | 30 | 47 | 87 | 21 | 20 | 17 | 31 | 95 | **24** | 31 | 28 | 42 | 85 | 23 | 26 | 26 | 34 | 88 |
| **8** Å | 27 | 31 | 28 | 47 | 86 | 19 | 19 | 16 | 30 | 95 | 23 | 31 | 28 | 40 | 84 | 23 | 26 | 26 | 35 | 87 |
| **9** Å | 27 | 31 | 27 | 47 | 86 | 17 | 17 | 15 | 28 | 95 | **24** | 31 | 28 | 43 | 83 | 23 | 26 | 25 | **36** | 87 |
| **10** Å | 25 | 29 | 26 | 45 | 86 | 17 | 16 | 15 | 28 | 94 | **24** | 32 | 27 | 46 | 82 | 21 | 25 | 24 | 35 | 86 |
| **11** Å | 22 | 26 | 24 | 41 | 85 | 10 | 10 | 9 | 18 | 95 | 23 | 31 | 28 | 44 | 81 | 20 | 23 | 22 | 34 | 86 |
| **12** Å | 21 | 25 | 23 | 39 | 85 | 10 | 10 | 8 | 19 | 94 | 22 | 31 | **29** | 43 | 81 | 19 | 23 | 22 | 32 | 86 |
| **13** Å | 19 | 24 | 22 | 37 | 85 | 9 | 9 | 7 | 18 | 94 | 23 | **33** | 27 | **51** | 78 | 18 | 22 | 22 | 32 | 86 |
| **14** Å | 16 | 21 | 19 | 33 | 85 | 8 | 8 | 6 | 16 | 94 | 20 | 31 | 26 | 47 | 78 | 15 | 19 | 19 | 28 | 86 |
| **15** Å | 14 | 19 | 17 | 31 | 85 | 6 | 6 | 5 | 13 | 94 | 20 | 31 | 25 | 50 | 77 | 13 | 18 | 17 | 26 | 85 |
| **16** Å | 12 | 17 | 15 | 28 | 85 | 5 | 5 | 4 | 11 | 94 | 19 | 30 | 24 | 49 | 76 | 12 | 16 | 15 | 25 | 85 |
| **17** Å | 9 | 14 | 12 | 23 | 85 | 3 | 3 | 3 | 7 | 95 | 17 | 28 | 24 | 43 | 77 | 9 | 13 | 12 | 21 | 85 |
| **18** Å | 7 | 11 | 10 | 18 | 86 | 2 | 2 | 1 | 4 | 95 | 16 | 27 | 24 | 43 | 75 | 7 | 11 | 9 | 17 | 86 |
| **19** Å | 7 | 12 | 10 | 21 | 85 | 2 | 2 | 1 | 4 | 95 | 17 | 28 | 23 | 49 | 73 | 7 | 11 | 10 | 19 | 84 |
| **20** Å | 6 | 10 | 8 | 18 | 85 | 1 | 1 | 1 | 2 | 96 | 16 | 28 | 23 | 45 | 74 | 5 | 9 | 8 | 17 | 85 |

MCC, F1, Precision, Recall, and accuracy on the DevSet1010 for structure cutoffs from 1-20Å of the GCNConv model are shown and rounded to the nearest number. For all models, predicted AlphaFold 2^21^ structures and DSSP^22^ features were used for the reported performances. The performances from predicting DevSet1010 and using the consensus prediction over all five models are shown. Except for nuclear predictions, the model with only minimal structural information (at max 3Å, approximately the distance between consecutive C_α_ atoms) achieves the best performance. An increase in the structure cutoff to include more distant amino acids leads to a drop in predictive performance.

For each column, numerically highest performances are highlighted in bold.

##### Table S21: Validation performances of SAGEConv on different distance cutoffs for DevSet1010.

| **Distance Cutoff** | **Overall** | | | | | **Metal** | | | | | **Nuclear** | | | | | **Small** | | | | |
| --- | --- | --- | --- | --- | --- | --- | --- | --- | --- | --- | --- | --- | --- | --- | --- | --- | --- | --- | --- | --- |
|  | MCC | F1 | Prec | Recall | Acc | MCC | F1 | Prec | Recall | Acc | MCC | F1 | Prec | Recall | Acc | MCC | F1 | Prec | Recall | Acc |
| **1** Å | 39 | 41 | 42 | 51 | **90** | 29 | 29 | 30 | 31 | **98** | 18 | 21 | 21 | 23 | **92** | 27 | 29 | 33 | 32 | **92** |
| **2** Å | 40 | 42 | 42 | 53 | **90** | 32 | 32 | 35 | 33 | **98** | 20 | 23 | 24 | 26 | **92** | 28 | 30 | 32 | 34 | 91 |
| **3** Å | 39 | 42 | 42 | 53 | **90** | 30 | 30 | 32 | 31 | **98** | 20 | 23 | 24 | 26 | 91 | 28 | 30 | 32 | 34 | 91 |
| **4** Å | 39 | 42 | 41 | 54 | **90** | 30 | 30 | 32 | 32 | **98** | 22 | 26 | 27 | 31 | 90 | 28 | 30 | 31 | 36 | 91 |
| **5** Å | 40 | 43 | 42 | 54 | **90** | 31 | 31 | 33 | 33 | **98** | 24 | 28 | 27 | 33 | 90 | 29 | 31 | 32 | 36 | 91 |
| **6** Å | 41 | 44 | 42 | 55 | **90** | 33 | 32 | 34 | 35 | **98** | 26 | 30 | 29 | 36 | 89 | 30 | 32 | 34 | 38 | 91 |
| **7** Å | 39 | 42 | 41 | 54 | 89 | 30 | 29 | 31 | 32 | **98** | 24 | 29 | 29 | 34 | 88 | 28 | 30 | 32 | 36 | 90 |
| **8** Å | **42** | **45** | 43 | **56** | **90** | **34** | **34** | **36** | **36** | **98** | 29 | 34 | 32 | 41 | 89 | **31** | 33 | 35 | **40** | 90 |
| **9** Å | 40 | 43 | 43 | 53 | **90** | 32 | 31 | 34 | 33 | **98** | 27 | 31 | 31 | 37 | 88 | 30 | 32 | 34 | 37 | 91 |
| **10** Å | **42** | 44 | **44** | 55 | **90** | **34** | **34** | **36** | **36** | **98** | 30 | 35 | **34** | 43 | 88 | **31** | 33 | 35 | 39 | 91 |
| **11** Å | 40 | 43 | 42 | 55 | **90** | 33 | 32 | 35 | 34 | **98** | 29 | 34 | 33 | 41 | 88 | 30 | 33 | 33 | 39 | 90 |
| **12** Å | 40 | 43 | 43 | 53 | **90** | 32 | 32 | 34 | 33 | **98** | 28 | 33 | 31 | 41 | 87 | 30 | 32 | 34 | 38 | 90 |
| **13** Å | **42** | 44 | 43 | **56** | **90** | 33 | 33 | 34 | **36** | **98** | 31 | **37** | 33 | 46 | 87 | **31** | **34** | 35 | **40** | 90 |
| **14** Å | **42** | 44 | **44** | 55 | **90** | 33 | 33 | 34 | **36** | **98** | 31 | 36 | 33 | 46 | 87 | **31** | 33 | **36** | 39 | 91 |
| **15** Å | 41 | 44 | 43 | 55 | **90** | 32 | 32 | 34 | 34 | **98** | 30 | 35 | 33 | 46 | 87 | 30 | 33 | 34 | 39 | 90 |
| **16** Å | 40 | 43 | 43 | 54 | 89 | 31 | 31 | 33 | 33 | **98** | 29 | 35 | 32 | 44 | 86 | 30 | 32 | 34 | 38 | 90 |
| **17** Å | 40 | 43 | 43 | 54 | **90** | 32 | 32 | 34 | 33 | **98** | 31 | 36 | **34** | 45 | 87 | 30 | 32 | 34 | 38 | 90 |
| **18** Å | 40 | 43 | 43 | 55 | 89 | 32 | 32 | 34 | 33 | **98** | 29 | 34 | 31 | 45 | 86 | 30 | 32 | 34 | 39 | 90 |
| **19** Å | 41 | 43 | 43 | 54 | **90** | 32 | 32 | 34 | 33 | **98** | 31 | 36 | 33 | 46 | 87 | 30 | 32 | 34 | 38 | 90 |
| **20** Å | 41 | 44 | 43 | 55 | **90** | 33 | 33 | 35 | 35 | **98** | **32** | **37** | 33 | **48** | 86 | 30 | 32 | 34 | 38 | 90 |

MCC, F1, Precision, Recall, and accuracy on the DevSet1010 for structure cutoffs from 1-20Å of the SAGEConv model are shown, rounded to the nearest number. For all models, predicted AlphaFold 2^21^ structures and DSSP^22^ features were used for the reported performances. The performances from predicting DevSet1010 and using the consensus prediction over all five models are shown. Increasing the structure cutoff to include more distant amino acids generally improves binding residue prediction performance up to a cutoff of 10Å. Further increase of the structure cutoff beyond this value does not show significant improvements in performance anymore. The best performance for binary prediction of binding/non-binding and metal binding is achieved at 10Å. However, these performances deviate only slightly from the ones at e.g. 14Å. For nuclear binding the best performance is achieved at 20Å, and for small binding, at 13-14 Å

For each column, numerically highest performances are highlighted in bold.

##### Table S22: Validation performances of SAGEConvMLP on different distance cutoffs for DevSet1010.

| **Distance Cutoff** | **Overall** | | | | | **Metal** | | | | | **Nuclear** | | | | | **Small** | | | | |
| --- | --- | --- | --- | --- | --- | --- | --- | --- | --- | --- | --- | --- | --- | --- | --- | --- | --- | --- | --- | --- |
|  | MCC | F1 | Prec | Recall | Acc | MCC | F1 | Prec | Recall | Acc | MCC | F1 | Prec | Recall | Acc | MCC | F1 | Prec | Recall | Acc |
| **1** Å | 38 | 40 | 40 | **52** | 89 | 19 | 19 | 17 | 26 | 95 | 6 | 7 | 7 | 8 | 94 | 23 | 25 | 27 | 29 | 89 |
| **2** Å | 38 | 41 | 41 | **52** | 90 | 20 | 20 | 18 | 26 | 95 | 6 | 7 | 7 | 7 | 94 | 23 | 25 | 27 | 29 | 90 |
| **3** Å | 39 | 41 | 42 | 50 | 90 | 22 | 22 | 22 | 26 | 96 | 6 | 7 | 9 | 7 | **95** | 24 | 25 | 28 | 28 | 90 |
| **4** Å | 40 | 42 | 44 | 51 | 90 | 23 | 22 | 22 | 26 | 97 | 8 | 9 | 10 | 10 | **95** | 24 | 26 | 29 | 27 | 91 |
| **5** Å | 40 | 42 | 45 | 49 | 91 | 23 | 23 | 23 | 27 | 97 | 10 | 11 | 12 | 12 | **95** | 24 | 26 | 30 | 26 | **92** |
| **6** Å | 40 | 42 | 44 | 51 | 90 | 23 | 22 | 22 | 26 | 97 | 8 | 9 | 10 | 11 | **95** | 25 | 26 | 30 | 28 | **92** |
| **7** Å | 41 | 43 | 45 | 51 | 91 | 25 | 25 | 26 | 27 | 97 | 14 | 16 | 17 | 18 | 94 | 26 | 28 | 31 | 29 | **92** |
| **8** Å | 40 | 43 | 44 | 51 | 91 | 26 | 26 | 28 | 27 | 97 | 13 | 15 | 16 | 17 | 94 | 26 | 27 | 30 | 30 | **92** |
| **9** Å | 41 | 44 | 46 | 51 | 91 | 27 | 27 | 29 | 29 | **98** | 17 | 20 | 21 | 23 | 93 | 26 | 28 | 32 | 29 | **92** |
| **10** Å | 42 | 44 | 47 | 50 | 91 | 28 | 28 | 31 | 29 | **98** | 27 | 30 | 31 | 34 | 91 | 27 | 28 | 33 | 30 | **92** |
| **11** Å | 41 | 43 | **48** | 49 | **92** | 26 | 26 | 27 | 28 | 97 | 21 | 23 | 25 | 26 | 93 | 27 | 28 | **34** | 28 | **92** |
| **12** Å | 41 | 44 | 45 | **52** | 91 | 27 | 27 | 29 | 28 | **98** | 14 | 16 | 16 | 18 | 94 | 27 | 29 | 32 | **31** | **92** |
| **13** Å | 42 | 44 | **48** | 49 | **92** | 29 | 29 | 32 | **30** | **98** | **30** | **34** | **35** | **39** | 90 | 27 | 29 | **34** | 29 | **92** |
| **14** Å | 40 | 42 | 45 | 50 | 91 | 26 | 25 | 27 | 27 | 97 | 13 | 16 | 16 | 18 | 93 | 26 | 28 | 32 | 29 | **92** |
| **15** Å | 42 | 44 | 47 | 51 | 91 | 29 | 29 | 32 | 29 | **98** | 21 | 24 | 25 | 28 | 92 | 27 | 29 | 33 | 30 | **92** |
| **16** Å | 42 | 44 | 47 | 50 | 91 | **30** | **30** | **33** | 29 | **98** | 29 | 33 | 34 | 38 | 90 | 27 | 29 | 33 | 30 | **92** |
| **17** Å | 40 | 43 | 44 | 51 | 91 | 26 | 26 | 28 | 27 | **98** | 11 | 12 | 13 | 15 | 94 | 26 | 28 | 31 | 30 | **92** |
| **18** Å | **43** | **45** | **48** | **52** | 91 | **30** | **30** | 32 | **30** | **98** | 22 | 25 | 25 | 28 | 93 | **28** | **30** | **34** | **31** | **92** |
| **19** Å | 41 | 43 | 46 | 49 | 91 | 26 | 26 | 27 | 27 | 97 | 21 | 23 | 25 | 26 | 92 | 26 | 27 | 32 | 28 | **92** |
| **20** Å | 42 | 44 | 46 | 51 | 91 | 27 | 27 | 29 | 28 | **98** | 17 | 19 | 20 | 22 | 93 | 27 | 29 | 32 | **31** | **92** |

MCC, F1, Precision, Recall, and accuracy on the DevSet1010 for structure cutoffs from 1-20Å of the SAGEConvMLP model are shown, rounded to the nearest number. For all models, predicted AlphaFold 2^21^ structures and DSSP^22^ features were used for the reported performances The performances from predicting DevSet1010 and using the consensus prediction over all five models are shown. Overall, the models at a structure cutoff of 18Å show the highest performances in many of the provided metrics for all classes. Only for the nuclear class, prediction performance at 13Å is higher. Models with no or minimal structural information (1-3Å) perform significantly worse.

For each column, numerically highest performances are highlighted in bold.

##### Table S23: Validation performances of SAGEConvGATMLP on different distance cutoffs for DevSet1010.

| **Distance Cutoff** | **Overall** | | | | | **Metal** | | | | | **Nuclear** | | | | | **Small** | | | | |
| --- | --- | --- | --- | --- | --- | --- | --- | --- | --- | --- | --- | --- | --- | --- | --- | --- | --- | --- | --- | --- |
|  | MCC | F1 | Prec | Recall | Acc | MCC | F1 | Prec | Recall | Acc | MCC | F1 | Prec | Recall | Acc | MCC | F1 | Prec | Recall | Acc |
| **1** Å | 33 | 36 | 33 | 56 | 86 | 20 | 20 | 19 | 25 | 96 | 9 | 10 | 13 | 10 | 94 | 21 | 23 | 23 | 29 | 89 |
| **2** Å | 33 | 36 | 33 | 56 | 86 | 20 | 20 | 19 | 25 | 96 | 9 | 10 | 13 | 10 | 94 | 21 | 23 | 23 | 29 | 89 |
| **3** Å | 33 | 36 | 32 | 57 | 86 | 20 | 20 | 18 | 25 | 96 | 8 | 9 | 10 | 9 | 94 | 21 | 23 | 23 | 29 | 89 |
| **4** Å | 34 | 37 | 34 | 54 | 87 | 20 | 20 | 18 | 26 | 96 | 11 | 12 | 15 | 11 | 94 | 22 | 24 | 25 | 30 | 90 |
| **5** Å | 35 | 38 | 33 | 58 | 86 | 19 | 19 | 16 | 27 | 96 | 9 | 10 | 10 | 11 | 94 | 22 | 24 | 26 | 29 | 90 |
| **6** Å | 33 | 36 | 34 | 53 | 87 | 21 | 21 | 20 | 25 | **97** | 7 | 8 | 9 | 9 | **95** | 20 | 22 | 25 | 25 | 90 |
| **7** Å | 34 | 36 | 39 | 46 | **89** | 21 | 21 | 20 | 26 | **97** | **14** | **15** | **24** | 12 | 93 | 22 | 23 | **29** | 25 | **91** |
| **8** Å | 34 | 37 | 38 | 48 | **89** | 22 | 21 | 20 | 27 | **97** | 9 | 11 | 12 | 10 | **95** | 20 | 21 | 28 | 22 | **91** |
| **9** Å | 35 | 38 | 36 | 53 | 88 | 20 | 20 | 18 | 27 | 96 | 11 | 13 | 15 | 13 | 94 | 22 | 24 | **29** | 27 | **91** |
| **10** Å | 37 | 39 | 36 | 58 | 87 | 21 | 20 | 18 | 28 | 96 | 10 | 12 | 12 | 14 | 94 | 24 | 26 | **29** | 31 | 90 |
| **11** Å | 36 | 39 | 36 | 55 | 88 | 20 | 20 | 18 | 27 | 96 | 13 | **15** | 17 | 14 | 94 | 23 | 25 | 28 | 29 | 90 |
| **12** Å | 36 | 39 | 36 | 56 | 87 | 20 | 20 | 17 | 28 | 96 | 13 | **15** | 18 | 14 | 94 | 24 | 26 | 27 | 31 | 90 |
| **13** Å | **38** | **41** | 37 | **59** | 88 | 21 | 20 | 18 | 29 | 96 | 10 | 12 | 11 | 14 | 94 | 25 | 27 | 29 | **33** | 90 |
| **14** Å | **38** | **41** | **40** | 53 | **89** | **24** | **24** | **22** | **30** | **97** | 12 | 14 | 13 | **16** | 93 | **26** | 27 | **34** | 28 | **91** |
| **15** Å | **38** | **41** | 37 | 58 | 88 | 22 | 22 | 20 | **30** | 96 | 9 | 11 | 10 | 14 | 93 | **26** | **28** | 31 | 32 | **91** |
| **16** Å | 37 | 40 | **40** | 51 | **89** | 22 | 21 | 19 | 28 | 96 | 11 | 12 | 13 | 12 | 94 | 24 | 25 | 32 | 27 | **91** |
| **17** Å | **38** | **41** | 37 | **59** | 88 | 23 | 23 | 21 | **30** | 96 | 8 | 10 | 8 | 13 | 93 | **26** | **28** | 31 | 32 | **91** |
| **18** Å | **38** | 40 | 39 | 55 | **89** | 23 | 23 | 21 | **30** | 96 | 10 | 11 | 11 | 14 | 93 | 25 | 27 | 32 | 29 | **91** |
| **19** Å | **38** | **41** | 38 | 57 | 88 | 22 | 21 | 18 | **30** | 96 | 10 | 11 | 11 | 14 | 94 | 25 | 27 | 31 | 30 | **91** |
| **20** Å | 37 | 40 | 38 | 55 | 88 | 22 | 22 | 20 | 29 | 96 | 9 | 11 | 10 | 13 | 94 | 25 | 26 | 31 | 29 | **91** |

MCC, F1, Precision, Recall, and accuracy on the DevSet1010 for structure cutoffs from 1-20Å of the SAGEConvGATMLP model are shown, rounded to the nearest number. For all models, predicted AlphaFold 2^21^ structures and DSSP^22^ features were used for the reported performances The performances from predicting DevSet1010 and using the consensus prediction over all five models are shown. Overall, the models at a structure cutoff of 14Å show the highest performances in many of the provided metrics for all classes. Only for the nuclear class, prediction performance at 7Å is higher. Models with no or minimal structural information (1-3Å) perform significantly worse.

For each column, numerically highest performances are highlighted in bold.

# References for Supporting Online Material

1 Littmann, M., Heinzinger, M., Dallago, C., Weissenow, K. & Rost, B. Protein embeddings and deep learning predict binding residues for various ligand classes. *Scientific Reports* **11**, 23916 (2021). <https://doi.org:10.1038/s41598-021-03431-4>

2 Santana, C. A. *et al.* GRaSP: a graph-based residue neighborhood strategy to predict binding sites. *Bioinformatics* **36**, i726-i734 (2020). <https://doi.org:10.1093/bioinformatics/btaa805>

3 Pedregosa, F. *et al.* Scikit-learn: Machine Learning in Python. *Journal of Machine Learning Research* **12**, 2825-2830 (2011).

4 Wu, Q., Peng, Z., Zhang, Y. & Yang, J. COACH-D: improved protein–ligand binding sites prediction with refined ligand-binding poses through molecular docking. *Nucleic Acids Research* **46**, W438-W442 (2018). <https://doi.org:10.1093/nar/gky439>

5 Cui, Y., Dong, Q., Hong, D. & Wang, X. Predicting protein-ligand binding residues with deep convolutional neural networks. *BMC Bioinformatics* **20**, 93 (2019). <https://doi.org:10.1186/s12859-019-2672-1>

6 Zeng, M. *et al.* Protein–protein interaction site prediction through combining local and global features with deep neural networks. *Bioinformatics* **36**, 1114-1120 (2020). <https://doi.org:10.1093/bioinformatics/btz699>

7 Jiménez, J., Doerr, S., Martínez-Rosell, G., Rose, A. S. & De Fabritiis, G. DeepSite: protein-binding site predictor using 3D-convolutional neural networks. *Bioinformatics* **33**, 3036-3042 (2017). <https://doi.org:10.1093/bioinformatics/btx350>

8 Xia, C.-Q., Pan, X. & Shen, H.-B. Protein–ligand binding residue prediction enhancement through hybrid deep heterogeneous learning of sequence and structure data. *Bioinformatics* **36**, 3018-3027 (2020). <https://doi.org:10.1093/bioinformatics/btaa110>

9 Bekar-Cesaretli, A. A. *et al.* Conservation of Hot Spots and Ligand Binding Sites in Protein Models by AlphaFold2. *Journal of Chemical Information and Modeling* **64**, 960-973 (2024). <https://doi.org:10.1021/acs.jcim.3c01761>

10 Xia, Y., Xia, C.-Q., Pan, X. & Shen, H.-B. GraphBind: protein structural context embedded rules learned by hierarchical graph neural networks for recognizing nucleic-acid-binding residues. *Nucleic Acids Research* **49**, e51-e51 (2021). <https://doi.org:10.1093/nar/gkab044>

11 Wang, W. *et al.* GraphPLBR: Protein-Ligand Binding Residue Prediction With Deep Graph Convolution Network. *IEEE/ACM Transactions on Computational Biology and Bioinformatics* **20**, 2223-2232 (2023). <https://doi.org:10.1109/TCBB.2023.3239983>

12 Xia, Y., Pan, X. & Shen, H.-B. LigBind: Identifying Binding Residues for Over 1000 Ligands with Relation-Aware Graph Neural Networks. *Journal of Molecular Biology* **435**, 168091 (2023). <https://doi.org:https://doi.org/10.1016/j.jmb.2023.168091>

13 Jiang, Z., Shen, Y.-Y. & Liu, R. Structure-based prediction of nucleic acid binding residues by merging deep learning- and template-based approaches. *PLOS Computational Biology* **19**, e1011428 (2023). <https://doi.org:10.1371/journal.pcbi.1011428>

14 Zhao, Y. *et al.* A Point Cloud Graph Neural Network for Protein–Ligand Binding Site Prediction. *International Journal of Molecular Sciences* **25**, 9280 (2024).

15 Chen, Y. C. *et al.* PPI-hotspotID for detecting protein–protein interaction hot spots from the free protein structure. *eLife* **13**, RP96643 (2024). <https://doi.org:10.7554/eLife.96643>

16 Cong, H., Liu, H., Cao, Y., Liang, C. & Chen, Y. Protein–protein interaction site prediction by model ensembling with hybrid feature and self-attention. *BMC Bioinformatics* **24**, 456 (2023). <https://doi.org:10.1186/s12859-023-05592-7>

17 Jeevan, K., Palistha, S., Tayara, H. & Chong, K. T. PUResNetV2.0: a deep learning model leveraging sparse representation for improved ligand binding site prediction. *Journal of Cheminformatics* **16**, 66 (2024). <https://doi.org:10.1186/s13321-024-00865-6>

18 Tubiana, J., Schneidman-Duhovny, D. & Wolfson, H. J. ScanNet: an interpretable geometric deep learning model for structure-based protein binding site prediction. *Nature Methods* **19**, 730-739 (2022). <https://doi.org:10.1038/s41592-022-01490-7>

19 Zhang, J. & Kurgan, L. SCRIBER: accurate and partner type-specific prediction of protein-binding residues from proteins sequences. *Bioinformatics* **35**, i343-i353 (2019). <https://doi.org:10.1093/bioinformatics/btz324>

20 Evteev, S. A., Ereshchenko, A. V. & Ivanenkov, Y. A. SiteRadar: Utilizing Graph Machine Learning for Precise Mapping of Protein–Ligand-Binding Sites. *Journal of Chemical Information and Modeling* **63**, 1124-1132 (2023). <https://doi.org:10.1021/acs.jcim.2c01413>

21 Jumper, J. *et al.* Highly accurate protein structure prediction with AlphaFold. *Nature* **596**, 583-589 (2021). <https://doi.org:10.1038/s41586-021-03819-2>

22 Kabsch, W. & Sander, C. Dictionary of protein secondary structure: pattern recognition of hydrogen-bonded and geometrical features. *Biopolymers* **22**, 2577-2637 (1983). <https://doi.org:10.1002/bip.360221211>

23 Elnaggar, A. *et al.* ProtTrans: Toward Understanding the Language of Life Through Self-Supervised Learning. *IEEE Transactions on Pattern Analysis and Machine Intelligence* **44**, 7112-7127 (2022). <https://doi.org:10.1109/TPAMI.2021.3095381>

24 Heinzinger, M. *et al.* ProstT5: Bilingual Language Model for Protein Sequence and Structure. *bioRxiv*, 2023.2007.2023.550085 (2023). <https://doi.org:10.1101/2023.07.23.550085>

25 Elnaggar, A. *et al.* Ankh: Optimized Protein Language Model Unlocks General-Purpose Modelling. *arXiv* (2023).

26 Lin, Z. *et al.* Evolutionary-scale prediction of atomic-level protein structure with a language model. *Science* **379**, 1123-1130 (2023). <https://doi.org:10.1126/science.ade2574>

27 Geffen, Y., Ofran, Y. & Unger, R. DistilProtBert: a distilled protein language model used to distinguish between real proteins and their randomly shuffled counterparts. *Bioinformatics* **38**, ii95-ii98 (2022). <https://doi.org:10.1093/bioinformatics/btac474>

28 Zhang, N. *et al.* OntoProtein: Protein Pretraining With Gene Ontology Embedding. *arXiv [q-bio.BM]* (2022).
